# A sensitive, accurate, and high-throughput gluco-oligosaccharide oxidase-based HRP colorimetric method for assaying lytic polysaccharide monooxygenase activity

**DOI:** 10.1186/s13068-022-02112-2

**Published:** 2022-02-10

**Authors:** Shuaishuai Wu, Juan Tian, Ning Xie, Muhammad Adnan, Juan Wang, Gang Liu

**Affiliations:** 1 College of Life Sciences and Oceanography, Shenzhen Key Laboratory of Microbial Genetic Engineering, Shenzhen University, Shenzhen, 518060 China; 2 College of Life Sciences and Oceanography, Shenzhen Key Laboratory of Marine Biotechnology and Ecology, Shenzhen University, Shenzhen, 518060 China

**Keywords:** Lytic polysaccharide monooxygenase, Enzyme activity assay, Gluco-oligosaccharide oxidase, Horse radish peroxidase, *Trichoderma reesei*, *Thielavia terrestris*, *Sarocladium strictum*

## Abstract

**Background:**

The AA9 (auxiliary activities) family of lytic polysaccharide monooxygenases (AA9 LPMOs) is a ubiquitous and diverse group of enzymes in the fungal kingdom. They catalyse the oxidative cleavage of glycosidic bonds in lignocellulose and exhibit great potential for biorefinery applications. Robust, high-throughput and direct methods for assaying AA9 LPMO activity, which are prerequisites for screening LPMOs with excellent properties, are still lacking. Here, we present a gluco-oligosaccharide oxidase (GOOX)-based horseradish peroxidase (HRP) colorimetric method for assaying AA9 LPMO activity.

**Results:**

We cloned and expressed a GOOX gene from *Sarocladium strictum* in *Trichoderma reesei*, purified the recombinant SsGOOX, validated its properties, and developed an SsGOOX-based HRP colorimetric method for assaying cellobiose concentrations. Then, we expressed two AA9 LPMOs from *Thielavia terrestris*, TtAA9F and TtAA9G, in *T. reesei*, purified the recombinant proteins, and analysed their product profiles and regioselectivity towards phosphoric acid swollen cellulose (PASC). TtAA9F was characterized as a C1-type (class 1) LPMO, while TtAA9G was characterized as a C4-type (class 2) LPMO. Finally, the SsGOOX-based HRP colorimetric method was used to quantify the total concentration of reducing lytic products from the LPMO reaction, and the activities of both the C1- and C4-type LPMOs were analysed. These LPMOs could be effectively analysed with limits of detection (LoDs) less than 30 nmol/L, and standard curves between the A_515_ and LPMO concentrations with determination coefficients greater than 0.994 were obtained.

**Conclusions:**

A novel, sensitive and accurate assay method that directly targets the main activity of both C1- and C4-type AA9 LPMOs was established. This method is easy to use and could be performed on a microtiter plate for high-throughput screening of AA9 LPMOs with desirable properties.

**Supplementary Information:**

The online version contains supplementary material available at 10.1186/s13068-022-02112-2.

## Background

Lytic polysaccharide monooxygenases (LPMOs) constitute a large class of copper-dependent enzymes that catalyse the oxidative cleavage of glycosidic bonds in recalcitrant polysaccharides, such as cellulose, chitin, and xylan, in the presence of an external electron donor, thereby reducing crystallinity and making these polymers more accessible to hydrolytic enzymes [1, 2]. LPMOs were originally classified as glycoside hydrolase family 61 endoglucanase, as they produced small amounts of reducing sugars while acting on cellulosic substrates [3]. However, a major breakthrough by Vaaje-Kolstad et al. revealed that these enzymes oxidatively cleaved rather than hydrolyzed the β-(1 → 4)-linked bonds in polysaccharides, which led to the discovery of a wide variety of LPMOs as well as their exact nomenclature and classification [4]. Currently, LPMOs are divided into eight auxiliary activity (AA) families in the carbohydrate active enzyme (CAZy) database: families AA9–AA11 and AA13–AA17 (www.cazy.org). Among them, AA9 family LPMOs are produced by fungi, active mainly against crystalline cellulose and exhibit potential applications in the secondary biorefinery industry [5–7]. As AA9 LPMOs are a diverse group of enzymes with varying catalytic efficiency, it is necessary to screen for LPMOs with higher activity levels to determine which can be efficiently used in secondary biorefineries.

For the large-scale screening of LPMOs with high enzymatic activities, it is first necessary to develop a convenient, sensitive, and high-throughput assay method. AA9 LPMOs oxidize β-1,4-glucan in two ways. Cl LPMOs (class 1) oxidize the pyranose ring of glucose moieties at the C1 position, generating a large amount of aldonic acids and a small amount of native cello-oligosaccharides, with only the native cello-oligosaccharides having a reducing end, while C4 LPMOs (class 2) oxidize at the C4 position, generating geminal diols and native cello-oligosaccharides, with both products having reducing ends [8–10]. Compared with cellulase action, the amount of reducing sugars released by AA9 LPMO action is generally too small to be accurately detected by traditional reducing sugar analysis methods, such as the 3,5-dinitrosalicylic acid (DNS) method [9–11]. To address this problem, advanced quantitative analysis methods based on high-precision equipment, such as high-performance anion exchange chromatography (HPAEC), ultrahigh-performance liquid chromatography (UHPLC), or chromatography combined with mass spectrometry, have been established [12–14]. Although these methods are accurate and can provide detailed insights into soluble product formation, they are expensive, time-consuming and labour-intensive [15]. In this context, some new methods for determining the activity of LPMOs have been developed. When LPMOs are incubated with an external electron donor such as ascorbic acid in the absence of a cellulose substrate, hydrogen peroxide is produced linearly, depending on the LPMO concentration. Thus, a fast and easy method for assaying LPMO activity was established by quantifying the formation of hydrogen peroxide with Amplex Red/horseradish peroxidase [16]. In another study, 2,6-dimethoxyphenol (2,6-DMP) and hydrocoerulignone were used as chromogenic substrates to assay LPMO activity [15, 17]. 2,6-DMP or hydrocoerulignone was converted to the coloured product coerulignone by LPMOs with H_2_O_2_ as a cosubstrate. Thus, LPMO activity was determined by measuring the absorbance of the product coerulignone. These two methods are sensitive, easy-to-use, and do not require expensive equipment; therefore, they can be adapted for high-throughput LPMO screening. However, they are based on two side activities, H_2_O_2-_generating activity and peroxidase-like activity, which may not correlate with LPMO activity on polymeric substrates such as cellulose.

Recently, reduced phenolphthalein (rPHP) (a chromogenic agent for blood tests used in forensic science), which has been proposed as an oligosaccharide mimic, was used as a chromogenic substrate for LPMO activity assays [18]. In the presence of a proper cosubstrate, colourless rPHP is converted to pink PHP by LPMO catalysis, and the LPMO activity can be analysed spectrophotometrically. This is also a very sensitive and potentially high-throughput method, although there is still some doubt about the correlation between rPHP oxidizing activity and cellulose oxidative lytic activity, because the cosubstrates perform differently in these two reactions [18]. Wang et al. developed a suitable method for assaying the activity of C1-type LPMOs that is based on the binding of Ni^2+^ to the carboxyl group of insoluble lytic products [11]. In this reaction, C1-type LPMOs catalyse the lytic oxidation of phosphoric acid swollen cellulose (PASC), producing insoluble aldonic acids that capture the solute Ni^2+^ and reduce its concentration. The decrease in Ni^2+^ concentration was quantified with pyrocatechol violet as a complexometric indicator, which is correlated with LPMO activity. Although this method is simple and the assayed activity is directly related to the natural activity of LPMOs, it has some drawbacks due to its limitation to only C1-type LPMO activity assay and a relatively higher error caused by measuring the decrease in Ni^2+^ concentration.

**Scheme 1:**
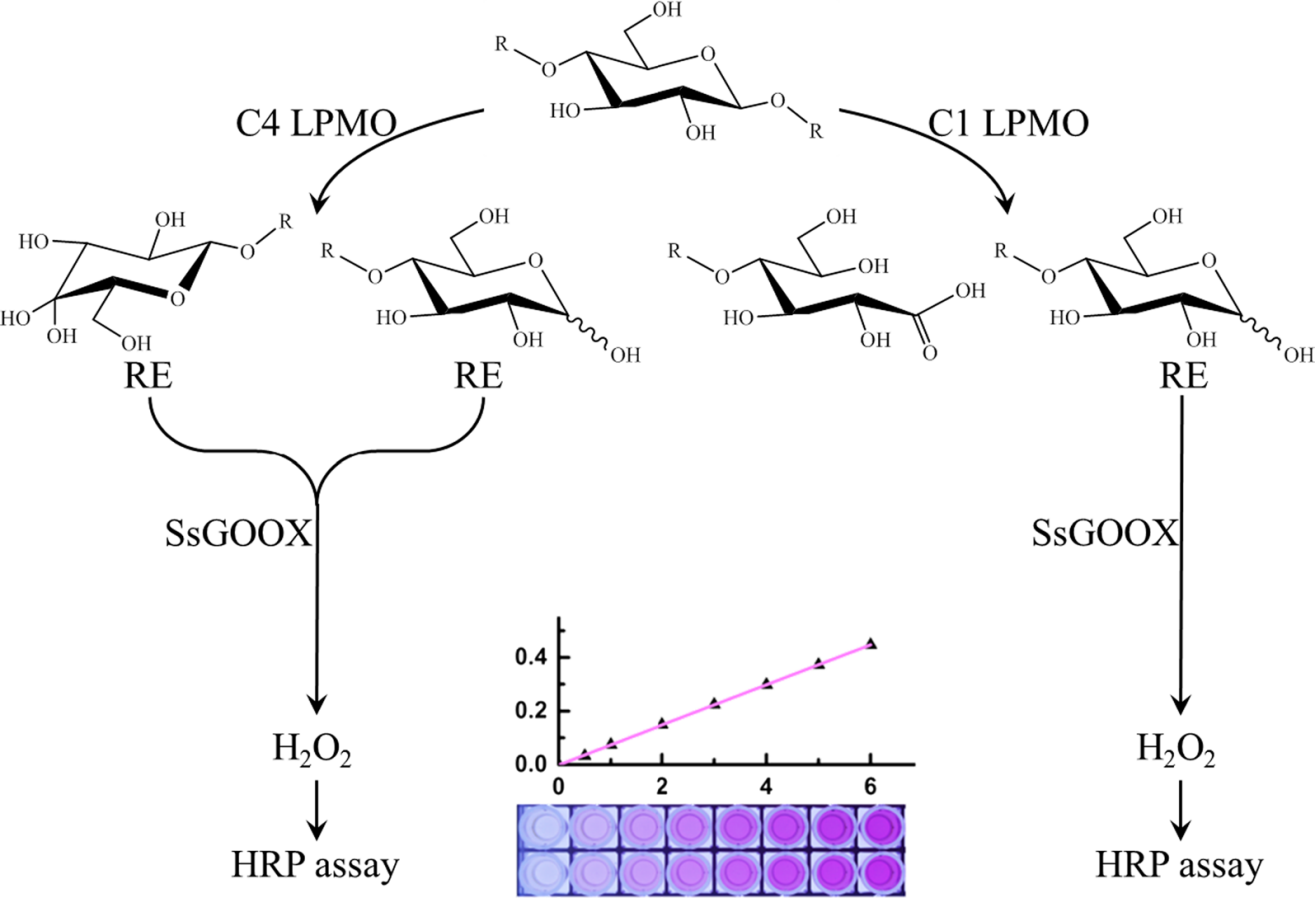
Schematic diagram of the GOOX-based HRP colorimetric method for assaying AA9 LPMO activity. The action of the C1-type LPMO towards PASC releases native oligosaccharides and aldonic oligosaccharide derivatives, with only native oligosaccharides having a reducing aldehyde end. The action of the C4-type LPMO releases native oligosaccharides and gemdiol oligosaccharide derivates, with both products having a reducing aldehyde end. The native oligosaccharides released by C1 LPMO cleavage and the two products released by C4 LPMO cleavage are then oxidized by GOOX, generating a stoichiometric amount of H_2_O_2,_ which is finally quantified by the HRP colorimetric assay. RE: reducing aldehyde end in oligosaccharides

Here, we designed a gluco-oligosaccharide oxidase (GOOX)-based method for assaying AA9 LPMO activity. GOOXs efficiently catalyse the oxidation of gluco-oligosaccharides, producing a stoichiometric amount of H_2_O_2_ that can be easily quantified by a horseradish peroxidase (HRP) colorimetric assay [19–22]. We expressed the GOOX gene from *Sarocladium strictum* (previously known as *Acremonium strictum* T1 [19]) in *Trichoderma reesei*, characterized the purified enzyme (SsGOOX) and developed an SsGOOX-based method for reducing sugars and assaying AA9 LPMO activity. Based on sequence alignment, a putative C1-type LPMO (sequence number THITE-2142696 in GenBank, designated TtAA9F in this work) and a putative C4-type LPMO (sequence number THITE-170174, designated TtAA9G in this work) from *Thielavia terrestris* were expressed in *T. reesei*. The C1- and C4-type LPMOs were purified, regioselectivity characterized, and used in the activity assay. The AA9 LPMO activity assay method is provided in Scheme 1. The reaction mixture for the AA9 LPMO activity assay contained PASC, LPMO and ascorbic acid (Asc). Upon LPMO action, reducing sugars were released, and after a certain period of time, ascorbic acid oxidase was added to exhaust the Asc and terminate the LPMO reaction. The reducing sugars were subsequently oxidized by adding SsGOOX, leading to equimolar production of H_2_O_2_, which is quantified by the HRP colorimetric assay.

## Results

### Heterologous expression and purification of SsGOOX

**Fig. 1:**
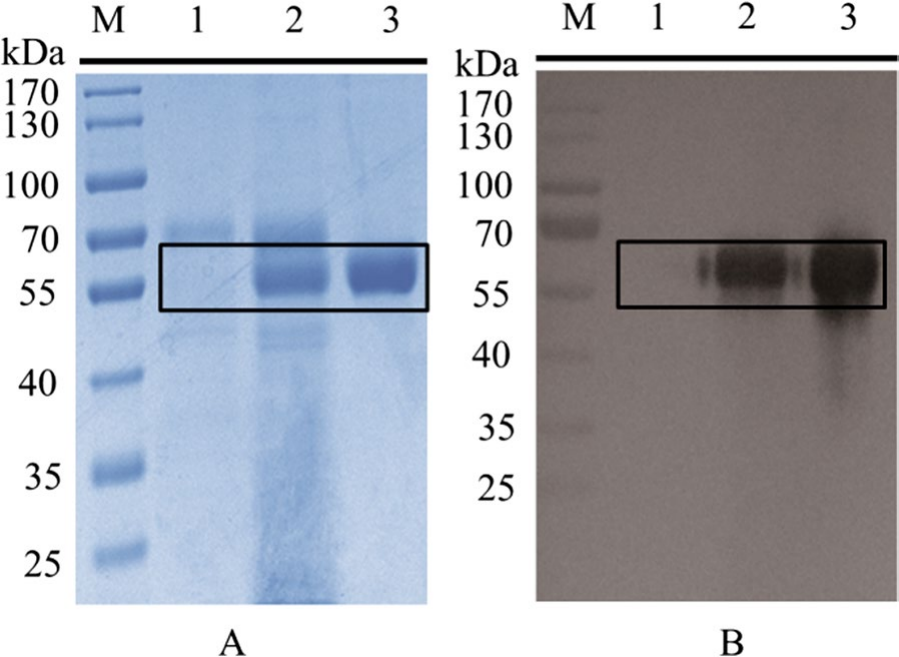
SDS–PAGE (**A**) and Western blot (**B**) analysis of the recombinant SsGOOX protein. M: molecular marker; **1** culture supernatant of the parent strain *T. reesei* QM9414; **2** culture supernatant of the recombinant strain; **3** purified recombinant SsGOOX

The mature peptide region of the SsGOOX gene encodes a 474 amino acid protein with a flavin adenine dinucleotide (FAD)-binding domain. It has a theoretical molecular weight of approximately 52.5 kDa and a theoretical pI (isoelectric point) of 4.66. The *T. reesei* transformants were verified by PCR amplification, DNA sequencing and Western blot analysis for the secreted recombinant protein using His-tag antibody. The selected transformant for recombinant SsGOOX production was cultured in 50 ml recombinant protein production medium for 6 days. After harvesting, the recombinant SsGOOX was purified to homogeneity using Ni^2+^-affinity chromatography and gel filtration chromatography. SDS–PAGE and Western blot analysis of the culture supernatant and purified SsGOOX are shown in Fig. 1. The productivity of recombinant SsGOOX was 184.8 mg/L as estimated by the densitometry method. The SDS–PAGE results confirmed that the molecular weight of the recombinant SsGOOX protein is similar to its theoretical value.

### Properties of the recombinant SsGOOX protein

**Fig. 2:**
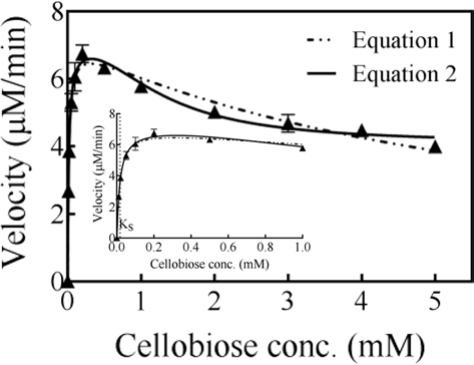
Simulation of the experimental data using the standard substrate inhibition model (the dashed line) and the modified Hill’s model (the solid line). The data in low substrate range is magnified and depicted in the inset, in which the value of K_*S*_ is indicated by the dashed vertical line. Error bars indicate the standard deviation (*n* = 3; independent experiments)

The properties of the purified recombinant SsGOOX were studied with cellobiose as the substrate. The HRP colorimetric assay was used to monitor the production of H_2_O_2_, with 4-amino-antipyrine (4-AAP) and 3, 5-dichloro-2-hydroxybenzenesulfonic acid (DCHBS) as chromogenic substrates [23]. Upon the oxidation of cellobiose by SsGOOX, a stoichiometric amount of H_2_O_2_ was produced, which was used by HRP to convert 4-AAP and DCHBS into a pink substance with maximum absorbance at 515 nm (A_515_). The concentrations of the chromogenic substrates 4-AAP and DCHBS used in the HRP colorimetric assay were determined to be 0.1 mM and 2.0 mM, respectively, based on the dependence of A_515_ on the 4-AAP and DCHBS concentrations (Additional file 1: Fig. S1). The kinetic parameters of the SsGOOX oxidation of cellobiose were correlated with the substrate concentration dependence on velocity. Similar to the results of Vuong et al. [21], the catalytic reaction of the recombinant SsGOOX towards cellobiose exhibited substrate inhibition (Fig. 2). We simulated the recombinant SsGOOX reaction towards cellobiose with both the standard substrate inhibition model (Eq. 1) and a modified Hill’s model (Eq. 2) [24], where K_*S*_ is the dissociation constant for substrate binding, *V*_max_ is the maximum reaction velocity, *V*_i_ is the reaction velocity in the presence of inhibition, K_*i*_ is the substrate inhibition constant, and *n*_H_ is the Hill coefficient:

1 $$v = \frac{{V_{\max } \cdot \left[ S \right]}}{{{\text{K}}_{s} + \left[ S \right] + \frac{{\left[ S \right]^{2} }}{{{\text{K}}_{i} }}}}$$

2 $$v = \frac{{V_{\max } + V_{i} \cdot \left( {\frac{{\left[ S \right]^{2} }}{{K_{i}^{2} }}} \right)}}{{1 + \frac{{{\text{K}}_{S}^{{n_{{\text{H}}} }} }}{{\left[ S \right]^{{n_{{\text{H}}} }} }} + \frac{{\left[ S \right]^{2} }}{{{\text{K}}_{i}^{2} }}}}$$

The initial parameters were set according to the parameters determined by Vuong et al. [21]: *V*_max_: 6.75 μM/min; V_*i*_: 2.16 μM/min; K_*i*_: 2.51 mM; K_*S*_: 0.048 mM; and *n*_H_: 2.2. As shown in Fig. 2, the modified Hill’s model simulated the experimental data better than the standard substrate inhibition model, with a coefficient of determination (*R*^2^ value) of 0.995. Therefore, the kinetic parameters were determined using the modified Hill’s model and are listed in Additional file 1: Table S1. The determined k_cat_, K_*S*_ and K_*i*_ were 729 min^−1^, 0.0177 mM and 0.995 mM, respectively, which differ from but are still comparable to those (k_*cat*_ = 420 min^−1^, K_*S*_ = 0.048 mM, K_*i*_ = 2.51 mM) determined by Vuong et al. [21]. The differences between the parameter values in this work and Vuong et al. might be due to the different approaches applied for parameter correlation. Vuong et al. first determined the K_*S*_ and *V*_max_ values by simulating the experimental data in the low-substrate-concentration region of the curve with the Michaelis–Menten equation and then determined the other parameters using the modified Hill’s equation. In this work, we directly determined all the parameters by simulating the experimental data with the modified Hill’s equation. Other reasons for the difference in the kinetic parameter values may be the use of different host strains for recombinant SsGOOX production and different pH values for determination of the enzyme reaction velocity in the kinetic study. For example, *P. pastoris* was used by Vuong et al. while *T. reesei* was used in this work; they assayed the enzyme reaction velocities at a pH of 8.0 buffered with Tris–HCl, while we did at a pH of 7.0 buffered with phosphate to accommodate with LPMO reaction [21].

During the pH effect and temperature effect experiments, the concentration of cellobiose was set at 0.1 mM to ensure an excess of substrate (higher than the K_*S*_ value). The effects of temperature and pH on the recombinant SsGOOX are shown in Additional file 1: Fig. S2. The optimal temperature of the recombinant SsGOOX was 50 °C, which is consistent with previously reported results [19]. Because the recombinant SsGOOX retains approximately 70% of its maximal activity at 30 °C, for ease of handling, in the following assays, the reaction was carried out at room temperature (approximately 26 °C). However, the recombinant SsGOOX has an optimum pH of 9.0, while the native GOOX of *Acremonium strictum* T1 has an optimum pH of 10.0 [19]. Moreover, the activity of the recombinant SsGOOX was not considerably affected at pH values lower than 9.0. At pH values of 8.0 and 7.0, the recombinant SsGOOX retained 97% and 85% of its maximal activity, respectively, while at a pH of 10.0, it retained only 71% of its activity. We propose that the difference between the pH effects in our experiment and those of Lin et al. might be due to the different glycosylation patterns of proteins in *T. reesei* and *Acremonium strictum* T1. As SsGOOX was used in conjunction with HRP in the detection of cello-oligosaccharides, the pH value in the reaction system was controlled at 7.0 with phosphate buffer in the following assays. With respect to thermostability, SsGOOX is relatively stable at 50 and 55 °C, which means that it retains almost 100% of its activity after being incubated for an hour at these temperatures. However, a higher temperature, i.e., 60 °C, can result in rapid deactivation (Additional file 1: Fig. S3).

### Detection of reducing sugar

**Fig. 3:**
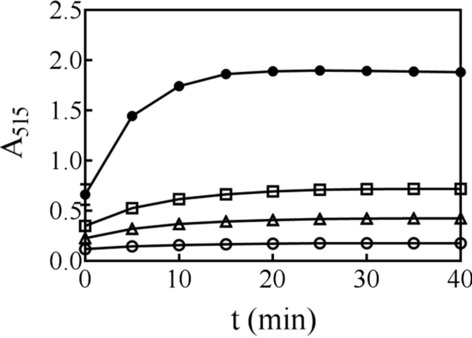
Cellobiose exhaustion upon recombinant SsGOOX oxidation over time indicated by HRP reaction. **○**: 0.01 mM; **△**: 0.04 mM;**□**: 0.08 mM;●: 0.32 mM. Error bars show standard deviation (*n* = 3; independent experiments)

We propose that SsGOOX is highly effective at oxidizing oligosaccharides under the appropriate reaction conditions. Indeed, under our reaction conditions, up to 0.32 mM cellobiose was completely oxidized within 25 min, as shown in Fig. 3. Initially, when the cellobiose concentration was low, A_515_ increased gradually over the first 25 min and then reached a maximum value that remained constant, indicating complete cellobiose exhaustion. In addition, when a solution containing 5 mg/ml cello-oligosaccharide mixture (G1 to G5, 1 mg/ml for each) standard was treated with 1 μM SsGOOX for 25 min, all the native oligosaccharides were converted into aldonic oligosaccharides, as revealed by HPAEC–PAD analysis (Additional file 1: Fig. S4). Therefore, the duration of the assay was determined to be 25 min based on the concentrations of cellobiose or oligosaccharides. When the initial cellobiose concentration was high (for example, 0.32 mM), the A_515_ value decreased slightly over time after reaching its peak value, possibly due to further oxidation of the pink substance by excessive H_2_O_2_. To observe this phenomenon, we manually added H_2_O_2_ and tested its effect on the stability of the pink substance, observing slight colour fading (data not shown).

**Fig. 4:**
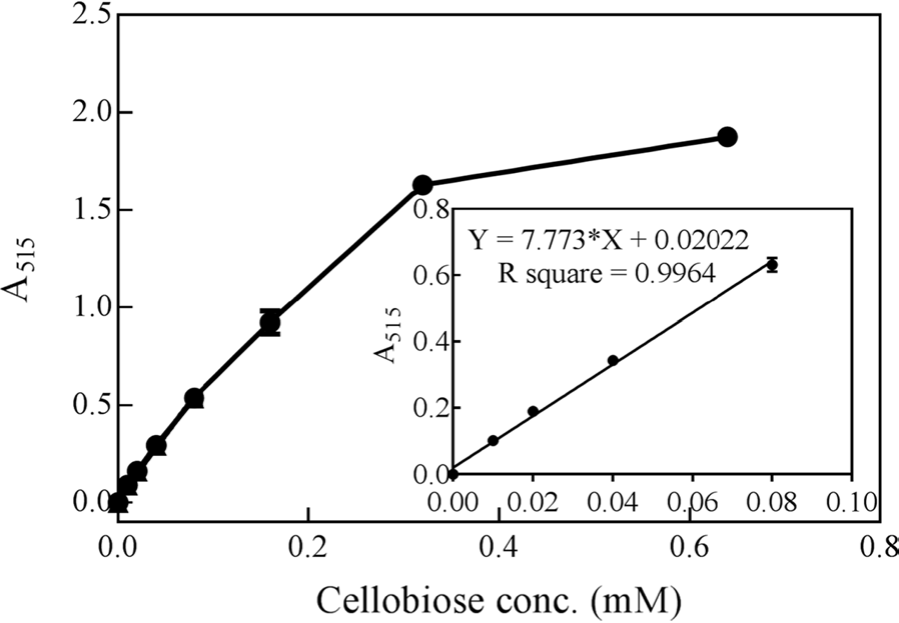
Relationship between A_515_ and cellobiose concentration in the SsGOOX-based HRP colorimetric cellobiose concentration assay. The standard curve of the low cellobiose concentration range is shown in the insert. Error bars show standard deviation (*n* = 3; independent experiments)

We determined the cellobiose concentration detection range for the SsGOOX-based assay. At low cellobiose concentrations (0–0.08 mM), the A_515_ value was proportional to the cellobiose concentration, and a standard curve with an *R*^2^ value of 0.996 was obtained (Fig. 4). In a broader cellobiose concentration range, the slope of the curve decreased in the high cellobiose concentration region, which might be due to the absorbance property of the pink substance, substrate inhibition, or further oxidation of the pink substance by excessive H_2_O_2_. To determine the lower limit of detection (LoD) of cellobiose, we assayed a series of cellobiose samples with low concentration, and found the lowest detectable concentration was 500 nmol/L. We thus assayed 60 independent diluted 500 nmol/L cellobiose samples and calculated the LoD for cellobiose assay according to Eq. (3) as suggested by Armbruster and Pry [25]. The LoD for cellobiose assay was determined as 993 nmol/L, close to 1 μmol/L:

3 $${\text{LoD}} = {\text{LoB}} + 1.645 \cdot \left( {{\text{SD}}_{{{\text{low}}\,{\text{concentration}}\,{\text{sample}}}} } \right)$$

where LoB was calculated according to the following equation:

4 $${\text{LoB}} = \,{\text{mean}}_{{{\text{blank}}}} + 1.645 \cdot \left( {{\text{SD}}_{{{\text{blank}}}} } \right)$$

### Heterologous expression and purification of LPMOs

**Fig. 5:**
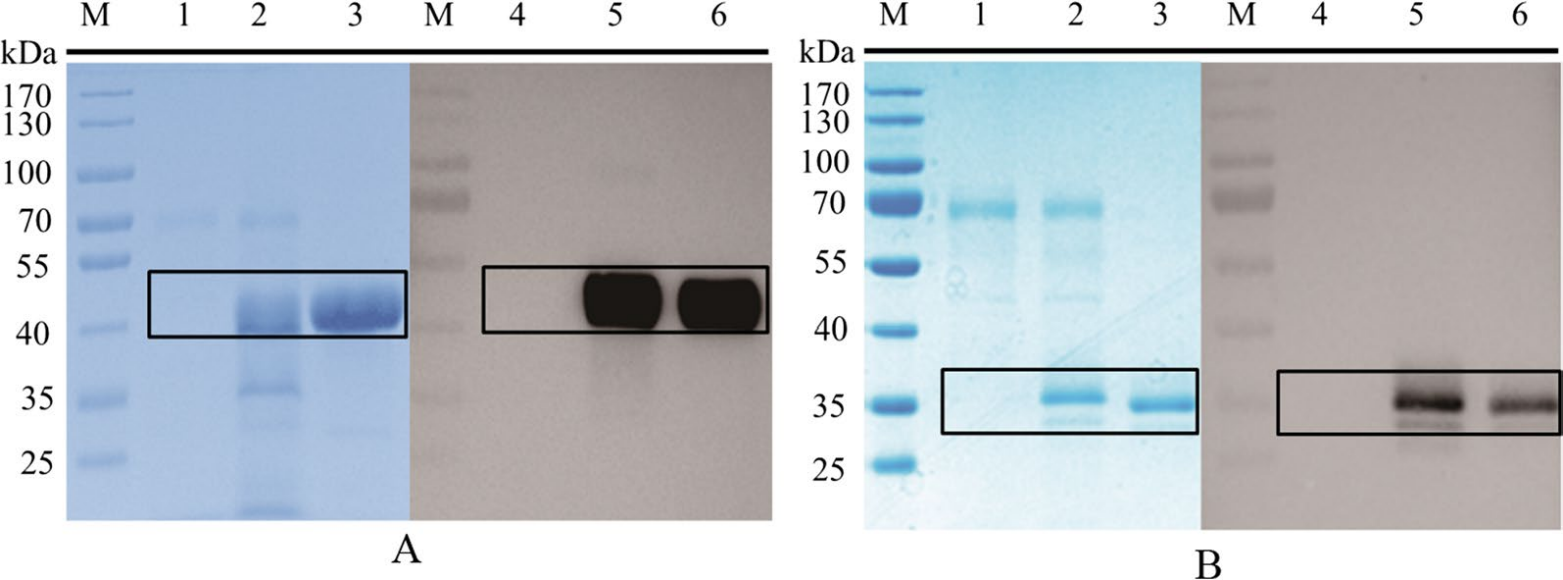
SDS–PAGE and Western blot analysis of recombinant TtAA9F (**A**) and recombinant TtAA9G (**B**). **M** molecular weight marker; **1** SDS–PAGE analysis of the culture supernatant of the parental strain; **2** SDS–PAGE analysis of the culture supernatant of the recombinant strain; **3** SDS–PAGE analysis of the purified recombinant protein; **4** Western blot analysis of the culture supernatant of the parental strain; **5** Western blot analysis of the culture supernatant of the recombinant strain; **6** Western blot analysis of the purified recombinant protein

LPMOs from *Thielavia terrestris* were among the earliest reported LPMOs and have been shown to boost cellulase activity towards lignocellulose deconstruction [26]; thus, we chose two LPMOs from *T. terrestris* to verify the applicability and reliability of the SsGOOX-based method for the LPMO activity assay. The coding sequences of these two LPMOs are THITE-2142696 and THITE-170174 in GenBank, and the corresponding LPMOs are designated TtAA9F and TtAA9G, respectively. The recombinant expression of the LPMOs in *T. reesei* culture supernatant was verified by SDS–PAGE and Western blot analysis (Fig. 5). TtAA9F contains cellulose-binding domain 1 (CBM1), while TtAA9G is CBM-free. The molecular weights of the recombinant TtAA9F and TtAA9G were approximately 40 kDa and 35 kDa, respectively, according to the SDS–PAGE analysis. The molecular masses according to the amino acid sequences of the recombinant proteins should be 32.5 kDa and 26 kDa, respectively. Therefore, glycosylation exists on both of the recombinant proteins of *T. reesei*. The concentrations of recombinant TtAA9F and TtAA9G in the culture supernatant were 240.8 mg/L and 155.7 mg/L, respectively, as estimated by the densitometry method. The recombinant proteins were both successfully purified to homogeneity with Ni^2+^-affinity chromatography and subsequent gel chromatography, as shown in Fig. 5.

### Activity and regioselectivity of the recombinant LPMOs

**Fig. 6:**
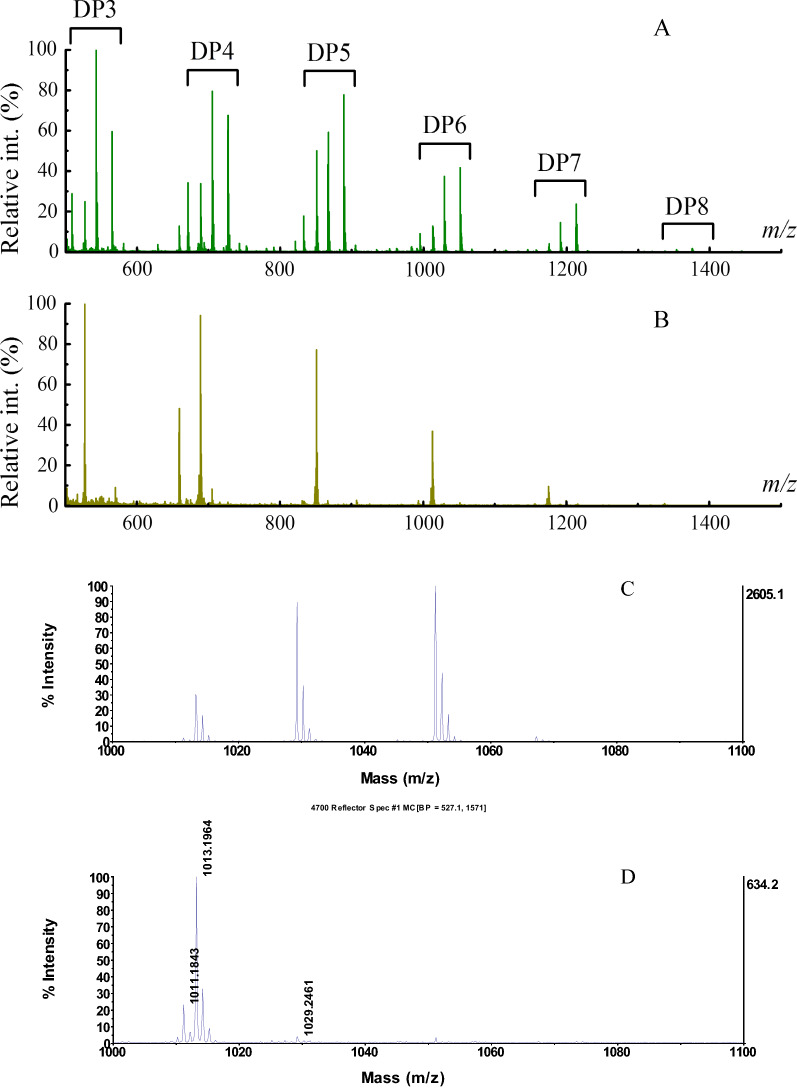
MALDI–TOF–MS analysis of the products generated by the two recombinant LPMOs with ascorbic acid as an electron donor. **A** Products generated by recombinant TtAA9F. **B** Products generated by recombinant TtAA9G. **C** Magnified DP6 peaks of (**A**); **D** Magnified DP6 peaks of (**B**)

The activities of TtAA9F and TtAA9G towards PASC using Asc as the electron donor were determined through matrix-assisted laser desorption/ionization time-of-flight mass spectrometry (MALDI–TOF–MS) analysis and high-performance anion exchange chromatography with pulsed amperometric detection (HPAEC–PAD). MALDI–TOF–MS analysis of the supernatant of the reaction mixture revealed that cello-oligosaccharides and their corresponding derivates were generated in the reactions; in the control reactions, no Asc was added, and thus, no cello-oligosaccharides or derivates were released. As shown in Fig. 6, the characteristic molecular ion peaks corresponding to cello-oligosaccharides or their derivates with various degrees of polymerization (DP) were present in the reaction supernatant of both LPMOs, indicating that the purified recombinant proteins were active LPMOs that acted in an endo-type oxidative cleavage mode. However, the two LPMOs produced distinctively different product profiles. As an example, the molecular ion peaks in the DP6 region were magnified and are shown in Fig. 6C, D. For TtAA9F, there were three main molecular ion peaks in this region: peak 1013, which corresponds to the sodium adduct of native hexasaccharide; peak 1029, which possibly corresponds to the sodium adduct of the aldonic acid form of hexasaccharide, the potassium adduct of native hexasaccharide, or the sodium adduct of the gemdiol form of hexasaccharide; and peak 1051, which possibly corresponds to the sodium adduct of the sodium salt of the aldonic form of hexasaccharide (Fig. 6C). Peak 1029 and peak 1051 dominate this region, indicating that TtAA9F acts in a C1 oxidative mode [27]. Therefore, upon TtAA9F action, PASC is oxidatively cleaved, generating a small amount of native cello-oligosaccharides and a large amount of aldonic acid derivatives of cello-oligosaccharides. The products of TtAA9G action have a significantly different molecular ion peak profile. As shown in Fig. 6D, there are two main molecular ion peaks in the DP6 region: peak 1013, which corresponds to the sodium adduct of hexasaccharide, and peak 1011, which corresponds to the sodium adduct of the ketoaldose derivative of hexasaccharide or the sodium adduct of the lactone derivative of hexasaccharide. The abundance of peak 1011 is 20% larger than that of peak 1013 in the product of TtAA9G, while this peak is negligible in the product of TtAA9F. Therefore, peak 1011 most likely corresponds to the sodium adduct of the ketoaldose derivative of hexasaccharide rather than the sodium adduct of the lactone derivative of hexasaccharide, because the hydrated gemdiol derivatives generated by C4 oxidation are more easily dehydrated as a result of sample preparation for MALDI–TOF–MS analysis than the aldonic acid derivatives generated by C1 oxidation. In addition, peak 1029 and peak 1051, which most likely correspond to the sodium adducts of aldonic acid derivates, are very small. Therefore, recombinant TtAA9G is most likely a C4-type LPMO that oxidatively cleaves PASC, releasing native cello-oligosaccharides and cello-oligosaccharide gemdiols.

**Fig. 7:**
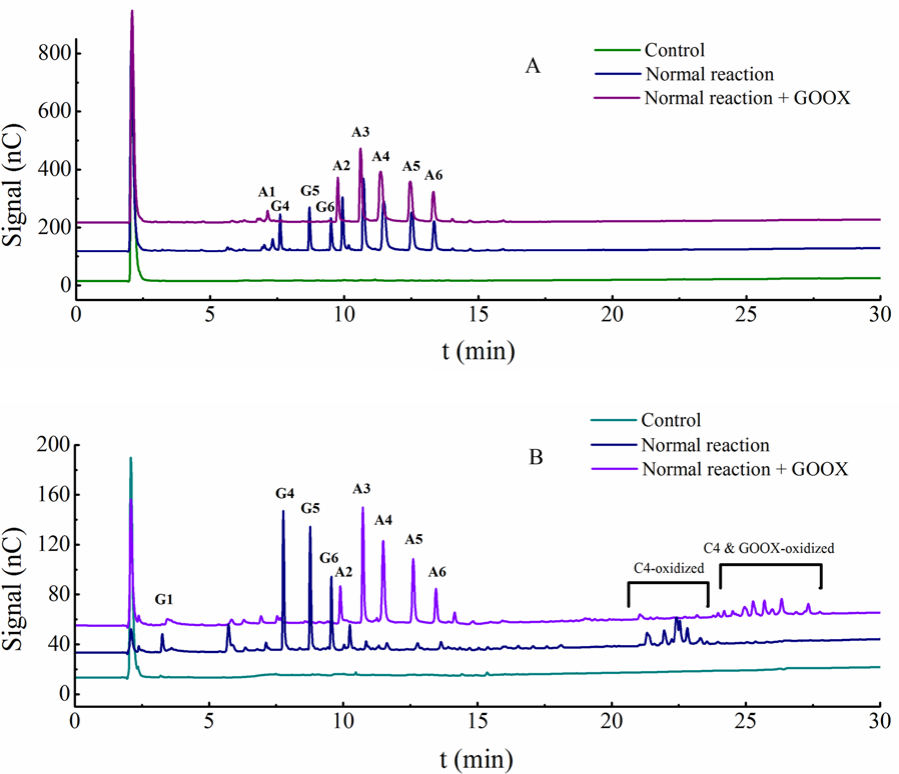
HPAEC–PAD analysis of the products generated by the reactions of TtAA9F (**A**) and TtAA9G (**B**) towards PASC. Control (lower line): control reaction without Asc addition; Normal reaction (middle line): LPMO reaction with Asc as the external donor; Normal reaction + GOOX (upper line): after the LPMO reaction, GOOX was added to oxidize the reducing sugars. Nonoxidized oligosaccharides from DP2 to DP5 and their corresponding aldonic acid oligosaccharides (oxidized at C1) were used as standards

We further identified the native cello-oligosaccharides and their oxidative derivates released by the reactions of TtAA9F and TtAA9G towards PASC with HPAEC–PAD (Fig. 7). The samples for the HPAEC–PAD assay were prepared in three different ways: (i) sample prepared with LPMO reaction using Asc as an electron donor (the middle blue line); (ii) sample prepared with LPMO reaction without the addition of Asc (the lower green line); and (iii) sample prepared with LPMO reaction using Asc as an electron donor, with the lytic products further oxidized by SsGOOX (the upper purple line). The products generated by TtAA9F were detected as a series of native cello-oligosaccharides with degrees of polymerization (DPs) from 4 to 6 and aldonic acid derivates of cello-oligosaccharides with DPs from 1 to 6. The cello-oligosaccharides were eluted at retention times ranging from 7.6 min to 9.5 min, while the aldonic acid derivates were eluted at retention times ranging from 7.3 min to 13.4 min. In the control reaction without Asc addition, no cello-oligosaccharides or oxidized derivates were released. When the cello-oligosaccharides and the oxidized derivates generated by the LPMO reaction were further oxidized by SsGOOX, the peaks corresponding to the native cello-oligosaccharides disappeared, while those corresponding to the C1-oxidized derivates of cello-oligosaccharide remained and slightly increased in the peak area (Fig. 7A). Therefore, the peak profile of the HPAEC–PAD analysis, as well as the change in the peak profile after SsGOOX oxidation, strongly suggested that TtAA9F was a C1-type LPMO.

For the products generated by TtAA9G, the peaks corresponding to native cello-oligosaccharides from DP1–DP6 were eluted at retention times ranging from 3.6 min to 9.6 min, while the peaks corresponding to the C4-oxidized oligosaccharide derivates were eluted at retention times ranging from 21.3 min to 23.3 min. There was no obvious formation of aldonic acid derivates of cello-oligosaccharide. When the products generated by TtAA9G were further oxidized by SsGOOX, the native cello-oligosaccharides were transformed into aldonic acid derivates, as indicated by the disappearance or emergence of the corresponding peaks, while the C4-oxidized cello-oligosaccharide derivates were transformed into 4-ketoaldonic acids or gemidiol aldonic acids, and the retention times of the corresponding peaks increased significantly (Fig. 7B). Therefore, based on the HPAEC–PAD analysis, TtAA9G was determined to be a C4-type LPMO that oxidatively cleaved PASC, releasing native cello-oligosaccharides and gemidiol derivates of cello-oligosaccharide, with both products having a reducing end.

### Removal of residual Asc with ascorbate oxidase

**Fig. 8:**
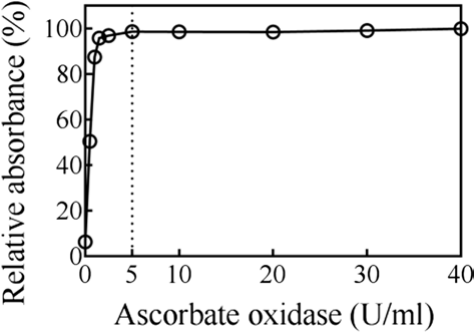
Elimination effect of ascorbate oxidase on residual Asc in SsGOOX-based HRP colorimetric assays. The reaction time for ascorbic acid oxidation was 1 min, the cellobiose concentration and Asc concentration for all of the tests were set at 0.1 mM and 1.0 mM, respectively, and the absorbance without ascorbic acid addition was defined as 100%. Error bars show standard deviation (*n* = 3; independent experiments)

Asc is a commonly used external electron donor for LPMO reactions. However, as previously reported, reducing agents such as Asc interfere with HRP colorimetric assays [28]. In our AA9 LPMO activity assay method, the HRP colorimetric assay was used to detect H_2_O_2_ generated by the oxidation of cello-oligosaccharides or their derivates by SsGOOX. Therefore, we first evaluated the effect of Asc on SsGOOX-based HRP colorimetric assays and discovered a way to eliminate this effect. As shown in Fig. 8, the presence of 1 mM Asc in the cellobiose detection system had a significant impact on the detection results (the first point represents the detected A_515_ value when there was no addition of ascorbate oxidase, which was only 6.25% of that of the control assay without Asc addition). Fortunately, the interference effects of Asc could be easily eliminated by ascorbate oxidase. After the addition of 5 U/ml or higher ascorbate oxidase and a 1 min reaction, the Asc in the cellobiose detection system was efficiently oxidized, and the detection results were similar to those of the control reaction without Asc (Fig. 8). To ensure the complete oxidization of the residual Asc, in the following assays, more ascorbate oxidase (20 U/ml) was used, and the reaction time for Asc oxidization was increased to 5 min.

### AA9 LPMO activity assay

**Fig. 9:**
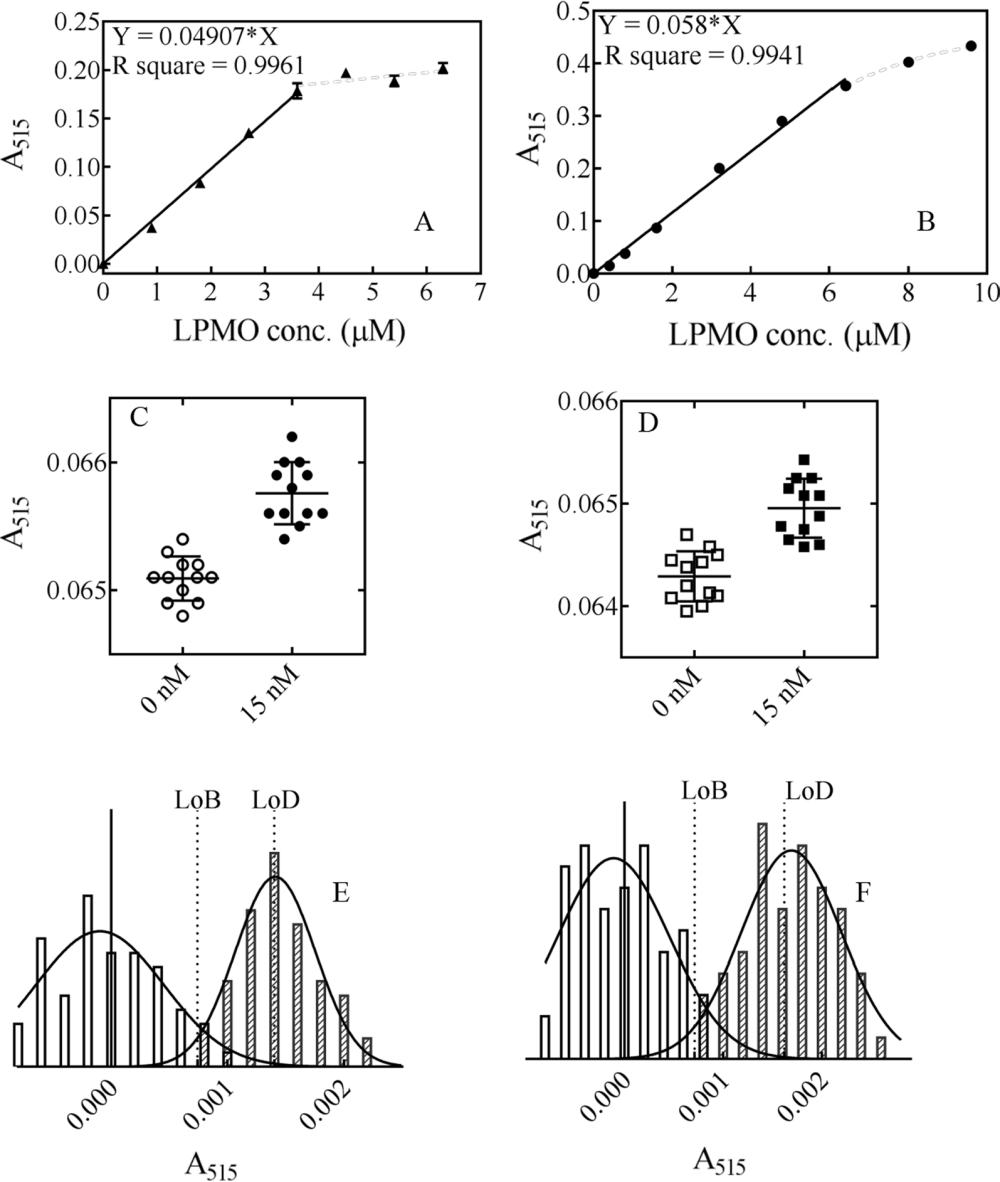
Detection of AA9 LPMO activity with the SsGOOX-based HRP colorimetric assay. **A** The A_515_-concentration curve of TtAA9F, with a linear relation found in the range 0–3.6 μM. **B** The A_515_-concentration curve of TtAA9G, with a linear range of 0–6.4 μM. **C** Comparison of the raw assay readings of 12 blanks and 12 independently diluted samples containing 15 nM TtAA9F. **D** Comparison of the raw assay readings of the blanks and the 15 nM TtAA9G samples. **E** Comparison of the LoB and 60 independently assayed results of the 28.6 nM TtAA9F samples. **F** Comparison of the LoB and 60 independently assayed results of the 27.9 nM TtAA9G at samples

For the AA9 LPMO activity assay, the GOOX-based cello-oligosaccharide detection method described above was used to detect the release of cello-oligosaccharides after LPMO action. The activities of both C1-type TtAA9F and C4-type TtAA9G were assayed. Different amounts of LPMO were added to the reaction mixture, which contained PASC as the substrate and Asc as the external electron donor, and the reaction mixture without Asc served as a control. After 30 min of incubation, the amount of reducing cello-oligosaccharides and their derivates released in the supernatant of the reaction mixture was analysed using the GOOX-based detection method. The amount of reducing sugars released was linearly correlated with the LPMO concentration (*R*^2^ > 0.994) when the concentrations of TtAA9F and TtAA9G were 0 to 3.6 μM and 0 to 6.4 μM, respectively (Fig. 9). The activities of the C1-type and C4-type LPMOs can both be analysed with the GOOX-based HRP colorimetric assay, and more importantly, this method directly targets the main activity of the AA9 LPMOs.

To determine the lower limit of detection (LoD), we assayed the activities of both LPMOs at a variety of low concentrations and found that the lowest detectable concentration for TtAA9F and TtAA9G was 15 nM (Fig. 9C, D). Therefore, the A_515_ readings with the deduction of the average blank A_515_ reading of 60 independently operated blanks without LPMO, 60 independently diluted and assayed 15 nM TtAA9F samples, and 60 independently diluted and assayed 15 nM TtAA9G samples were used to determine the LoDs of our method. The LoDs were calculated according to Eq. (3).

The LoBs (in the A_515_ reading and with deduction of the average blank A_515_ reading) for TtAA9F and TtAA9G were calculated as 0.000735 and 0.000713, respectively. The LoDs (in the A_515_ reading) for TtAA9F (C1-type) and TtAA9G were 0.00140 and 0.00162, respectively. The LoDs for the LPMO concentration for TtAA9F and TtAA9G were calculated to be 28.6 nM and 27.9 nM, respectively, according to the standard curves for LPMO activity analysis (Fig. 9A, B). The purified TtAA9F and TtAA9G samples were diluted to concentrations of 28.6 nM and 27.9 nM, respectively. Each dilution was independently repeated 60 times, and the diluted samples were assayed with our method and compared with the corresponding LoBs. The results revealed that all the assayed values were higher than the LoBs (Fig. 9E, F). Therefore, the LoDs of the C1-type LPMO and C4-type LPMO in the SsGOOX-based HRP colorimetric assay were verified to be 28.6 nM and 27.9 nM, respectively.

There is a notable difference between the ratios of the total cello-oligosaccharides to the reducing cello-oligosaccharides released by C1-type LPMOs and C4-type LPMOs: both the geminal diols and native cello-oligosaccharides generated by C4-type LPMOs have a reducing end, while only the native cello-oligosaccharides generated by C1-type LPMOs have a reducing end. Therefore, in principle, in our C4 LPMO activity assay, all the soluble products released can be detected, while in the C1 LPMO activity assay, only the native cello-oligosaccharides can be detected, leading to an underestimation of C1 LPMO activity. However, the underestimation of C1 LPMO activity can be corrected with the ratio of the amount of total soluble lytic products to the amount of native soluble cello-oligosaccharide released by the C1-type LPMO, which is designated as the T/R ratio in this work and was estimated to be 4.75 based on the peak area calculations in Fig. 7A. Although this coefficient may not be applicable to other C1-type LPMOs, it could provide a reference, or it could be independently determined for other C1-type LPMOs.

**Fig. 10:**
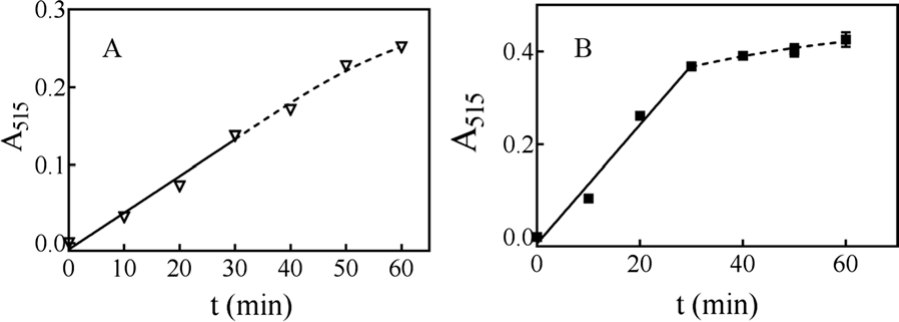
Determination of the initial speed range for the AA9 LPMO activity assay. **A** TtAA9F; **B** TtAA9G. Error bars show standard deviation (*n* = 3; independent experiments)

In a batch enzyme reaction, initially, the reaction speed is at its highest and is known as the “initial reaction speed”, which remains constant for a certain time; then, the reaction speed begins to decline due to substrate consumption, enzyme deactivation, or inhibitor accumulation. To obtain a more accurate analysis, the initial reaction time for LPMOs, during which the reaction speed remains constant, should be determined. For TtAA9F, when an enzyme concentration of 3.6 μM was used, the initial reaction time was 30 min, during which the reducing cello-oligosaccharide concentration increased linearly over time. For TtAA9G, the initial reaction time was also 30 min when an enzyme concentration of 6.4 μM was used (Fig. 10). Therefore, we assumed that a 30 min reaction time was appropriate for the AA9 LPMO activity assay.

We defined AA9 LPMO activity based on the analysis results; that is, one unit of AA9 LPMO activity was defined as the amount of enzyme that released one μmol of cello-oligosaccharides and their derivates in 1 min. According to this definition, the specific activity of TtAA9G was determined to be 0.122 U/mg, while the specific activity of TtAA9F was determined to be 0.270 U/mg when multiplied by the T/R ratio of 4.75. A clear definition of AA9 LPMO activity and specific activity is important for comparing the catalytic efficiencies of AA9 LPMOs of different origins.

### AA9 LPMO activity assay protocol

Based on the above results, we suggest the following protocol for assaying AA9 LPMO activity.

#### Step 1

Prepare the 196 μl LPMO reaction system in 1.5 ml Eppendorf tubes, which consists of 100 μl PASC suspension (with concentration of 90 g/L for the C1 LPMO activity assay and 30 g/L for the C4 LPMO activity assay), 10 μl LPMO solution, and 76 μl 100 mM buffer at its optimal pH (the optimal pH for TtAA9F and TtAA9G is approximately 7.5, and 100 mM phosphate buffer was used in this work). Set a reaction system with 10 μl pure water substituted for the 10 μl LPMO solution as a control. Pre-incubate the reaction mixture at 50 °C for 3 min, then start the reaction by adding 10 μl of 20 mM freshly prepared Asc and incubating at 50 °C for 30 min.

#### Step 2

Place the reaction mixture on ice immediately after removing it from the water bath, add 4 μl ascorbate oxidase solution (1000 U/ml) to the reaction mixture, and vortex the mixture for 5 min to remove excess Asc. Centrifuge the reaction mixture in a mini centrifuge at 12,000 r/min for 5 min, then transfer the supernatant to fresh tubes.

#### Step 3

Prepare the SsGOOX-based HRP colorimetric assay on a 96-well microtiter plate. The total reaction volume was 200 μl, which included 20 μl of the supernatant from the LPMO reaction, 20 μl of 1 mM 4-AAP, 20 μl of 20 mM DCHBS, 20 μl of 1 μM GOOX, 20 μl of 500 U/ml HRP and 100 μl of the sodium phosphate buffer (100 mM sodium phosphate, pH 7.0). The 4-AAP and DCHBS solutions should be stored separately and used within 12 h.

#### Step 4

Incubate the 96-well plate at room temperature for 25 min and then record the A_515_ on a microtiter plate reader.

#### Step 5

Calculate the total amount of reducing oligosaccharides or derivates released according to the pre-established A_515_-cellobiose concentration standard curve with the SsGOOX-based HRP colorimetric assay (for an example, see the insert of Fig. 4 in this work). For the C4 LPMO activity assay, the amount of total oligosaccharides or derivates equals the total amount of reducing oligosaccharides or derivates; for the C1 LPMO activity assay, the amount of total oligosaccharides or derivates equals the amount of reducing oligosaccharides times the T/R ratio. This ratio was determined to be 4.75 according to the HPAEC–PAD analysis results of the products released by TtAA9F. For other C1 LPMOs, the ratio can be determined based on the HPAEC–PAD analysis of that C1 LPMO.

## Discussion

A robust and easy method for assaying AA9 LPMO activity is important not only for screening AA9 LPMOs with high activity but also for studying their properties and catalytic mechanisms. Currently, there are two approaches for assaying LPMO activity. One approach is to determine the amount of cello-oligosaccharides or derivates released by the LPMO reaction through HPAEC–PID or HPLS–MS analysis [29–31]. This approach is time-consuming and has a low throughput. For example, analysing one sample using HPAEC usually takes 25 min. The second approach utilizes the side reaction activities that generate H_2_O_2_ or catalyse the oxidation of chromogenic substrates such as 2,6-DMP or rPHP to assess the LPMO activity using a colorimetric method [15, 16, 18]. Although this approach is potentially high-throughput, the assayed side reaction activities may not accurately correlate with the main activity that oxidatively cleaves the cellulosic substrate. Our work establishes a GOOX-based HRP colorimetric method for assaying AA9 LPMO activity that is accurate, sensitive, and potentially high-throughput, and, more importantly, directly targets the main activity of AA9 LPMOs.

GOOXs, which belong to the CAZy family AA7, catalyse the oxidation of gluco-oligosaccharides while simultaneously generating a stoichiometric amount of H_2_O_2_. Some GOOXs have proven to be highly effective towards gluco-oligosaccharides and can completely oxidize the substrate under proper reaction conditions [19–22]. Our results support the efficacy of GOOX action towards gluco-oligosaccharides and their corresponding derivates with a reducing end. After 30 min of SsGOOX treatment, the vast majority of cello-oligosaccharides or gemdiols were oxidized and transformed into their corresponding aldonic acids (Fig. 7). Therefore, GOOX oxidation coupled with the HRP colorimetric assay can be used to determine oligosaccharide concentration, cellulase activity, and LPMO activity. Ferrari et al. [32] developed a colorimetric method for assaying cellulase and chitinase activity with a mutant chito-oligosaccharide oxidase from *Fusarium graminearum*. Their assay outperformed the DNS method in terms of in sensitivity and ease of use. AA9 LPMOs catalyse the cleavage of crystalline cellulose, releasing a small amount of reducing sugars. In theory, analytic methods targeting reducing sugars, such as the DNS method, can be used to assay AA9 LPMO activity. However, the amount of reducing sugars released by AA9 LPMO is much smaller than that released by cellulase; therefore, the sensitivity of the DNS method is inadequate for an accurate assay. In contrast to the DNS method, the GOOX-based HRP colorimetric method is far more sensitive in detecting reducing sugars and thus exhibits great potential for accurate assays of reducing sugars released by AA9 LPMOs. We expressed a *S. strictum* GOOX gene in *T. reesei* using a strong constitutive *pdc* promoter and obtained purified recombinant SsGOOX [33]. Although there are minor differences, the properties of the recombinant SsGOOX are very similar to those of native or *P. pastoris*-expressed SsGOOX [21]. The purified recombinant SsGOOX was used in conjunction with the HRP colorimetric system for the cellobiose assay, and an A_515_-cellobiose concentration standard curve was obtained. The standard curve was comparable to that of Ferrari et al. [32]. To avoid complexity and maintain operational simplicity, we used cellobiose to establish the standard curve. SsGOOX has broad substrate specificity and catalyses the oxidation of cello-oligosaccharides with different DPs with kinetic parameters comparable to those of cellobiose [20]. This broad substrate specificity can also be verified by HPAEC–PAD analysis (as performed in this work, see Fig. 7 and Additional file 1: Fig. S4). Therefore, we suggest that the SsGOOX-based assay can detect all the soluble cello-oligosaccharides or their derivates with a reducing aldehyde group and can be used for cellulases or AA9 LPMO activity assays, where the units of activity are generally defined according to the rate of oligosaccharide release.

For the AA9 LPMO activity assay, Asc is frequently used as the external electron donor [29, 31, 34]. However, as a reducing reagent, Asc interferes with the HRP colorimetric detection results. Thus, we used ascorbate oxidase to successfully remove residual Asc; with the addition of a small amount of ascorbate oxidase (5 U/ml) and a short reaction time (less than 1 min), the interference of Asc on the HRP reaction was completely eliminated (Fig. 8). In addition, the efficient removal of Asc by ascorbate oxidase results in a fast termination of the LPMO reaction. Therefore, ascorbate oxidase serves two purposes in our SsGOOX-based HRP colorimetric assay: it eliminates Asc interference and quickly terminates the LPMO reaction. Another factor that interferes with HRP colorimetric detection is the futile formation of H_2_O_2_ during the LPMO reaction. In the presence of the reductant and the absence of the substrate, LPMO reactions produce H_2_O_2_, and this characteristic was used to quantify the activity of LPMOs by Kittl et al. [16]. In the presence of the substrate, substrate-bound LPMOs are generally assumed to be incapable of producing H_2_O_2_, but nonsubstrate-bound LPMOs still produce small amounts of H_2_O_2_ [35, 36]. The futile formation of H_2_O_2_ in both TtAA9F and TtAA9G reactions was detected using the HRP colorimetric assay (without oxidation of the reducing sugars by SsGOOX, see Additional file 1: Fig. S5). However, in our assay procedure, there was no obvious futile formation of H_2_O_2_, possibly because an excess amount of PASC was used in the LPMO reaction system and the reactions were performed in a relatively short reaction time (30 min) (Additional file 1: Fig. S5). A small amount of futile H_2_O_2_ was formed in TtAA9G reaction when the reaction time increased. A number of studies have demonstrated that H_2_O_2_ is an efficient co-substrate as compared to oxygen, and it is frequently added into LPMO reaction system to enhance the reaction efficiency [35, 36]. In these conditions, the residual H_2_O_2_ might interfere with the GOOX-based HPR colorimetric assay. In our experience, we have found that a proper amount of HRP can be used to eliminate the residual prior to GOOX based HPR colorimetric assay (data not shown).

Based on the sequence alignment analysis, we selected two AA9 LPMOs from *T. terrestris* for the enzyme activity assay. The first was TtAA9F, which was determined to be a C1-type LPMO by MALDI–TOF analysis and HPAEC–PAD analysis of the cleavage products released from PASC; the second was TtAA9G, which was determined to be a C4-type LPMO. It is worth noting that GOOX can also play an important role in the regioselectivity analysis of AA9 LPMOs. For the oxidative cleavage products of the AA9 LPMO reaction further treated with SsGOOX, the peak profile of the HPAEC–PAD analysis changed significantly, with different changes observed for the C1 oxidized products and C4 oxidized products (Fig. 7). By detecting the concentration of reducing cello-oligosaccharides and their derivatives, the SsGOOX-based HRP colorimetric method can detect concentrations as low as 28.6 nmol/L for C1-type LPMOs (for TtAA9F, 28.6 nmol/L equals 0.929 mg/L) and 27.9 nmol/L for C4-type LPMOs (for TtAA9G, 27.9 nmol/L equals 0.726 mg/L). The standard curves of A_515_ versus LPMO concentration have coefficients of determination that are greater than 0.994. Thus, we suggest that the SsGOOX-based HRP colorimetric method for assaying AA9 LPMO activity presented here has the following advantages: (i) after the LPMO reaction, the whole assay process can be conveniently operated on a microtiter plate, and the assay results can be recorded with a microtiter plate reader; therefore, it is a high-throughput assay method; (ii) the assay directly targets the main activity of AA9 LPMOs rather than the side activities; (iii) the activities of both C1- and C4-type LPMOs can be assayed; (iv) the method is sensitive and accurate; and (v) except for a microplate reader, no other expensive instrument is needed; thus, it is a cheap and easy-to-use method.

In the assay process, three tool enzymes were used: HRP, ascorbate oxidase, and SsGOOX. HRP and ascorbate oxidase are both commercially available, while SsGOOX was prepared in our lab. The HRP colorimetric system is a convenient and commonly used tool for detecting H_2_O_2_. Ascorbate oxidase was added immediately after the LPMO reaction, removing residual Asc while simultaneously terminating the LPMO reaction; thus, the Asc removal step does not add any complexity to the assay process. SsGOOX was added with the HRP colorimetric mixture; therefore, the addition of SsGOOX does not add any complexity to the assay process. If SsGOOX or other comparable GOOXs become commercially available, this GOOX-based AA9 LPMO activity assay method will be more convenient to use.

For the C1-type LPMO activity assay, the activity of the C1-type LPMOs might be underestimated, because the aldonic acids released cannot be detected by the SsGOOX-based HRP colorimetric assay. Therefore, a ratio of the amount of total soluble lytic products to the amount of native cello-oligosaccharides (T/R ratio) was introduced to compensate for this underestimation. The product of the LPMO reaction was sampled for the activity assay in the initial speed region, i.e., within 30 min of the reaction (Fig. 10). In the initial speed region of an enzymatic reaction, the reaction speed is generally assumed to be constant; thus, the formation of total oligosaccharides and their derivatives was linearly correlated with the reaction time. On the other hand, the formation of reducing oligosaccharides also has a linear correlation with the reaction time, as shown in Fig. 10. Therefore, the ratio of the amount of total oligosaccharides and their derivates to the amount of reducing oligosaccharides (T/R ratio) should be constant in the initial speed region and was determined to be 4.75 based on the HPAEC–PAD analysis results.

In the large-scale screening of AA9 LPMOs, the use of the HRP colorimetric method might be restricted to some extent if the samples are contaminated with endoglucanase. If the samples are derived from recombinant LPMOs produced by *Pichia pastoris* or filamentous fungi or native LPMOs produced by fungi, contamination of the host endoglucanases may occur. Because endoglucanases are more effective than LPMOs at releasing native oligosaccharides from amorphous cellulosic substrates such as PASC, the contaminant endoglucanases produce relatively large amounts of native oligosaccharides and thus interfere with the GOOX-based HRP colorimetric assay [9, 37]. However, this assay method is still applicable in cases where the LPMO samples are endoglucanase free, such as those derived from *E. coli*-expressed recombinant LPMOs or completely purified LPMO samples [38, 39].

## Conclusion

We have established a sensitive, robust, and direct gluco-oligosaccharide oxidase-based HRP colorimetric method for assaying AA9 LPMO activity. This method can detect the activities of both C1- and C4-type AA9 LPMOs at concentrations as low as 28.6 nmol/L and 27.9 nmol/L, respectively, targeting their main activities with a standard curve with an *R*^2^ value greater than 0.994. The assay process can be performed on ready-to-use microtiter plates for high-throughput screening and, therefore, has potential applications in the large-scale screening of AA9 LPMOs with high activity or in screening procedures for the directed evolution of AA9 LPMOs. Based on the assay method, we proposed a definition of the AA9 LPMO activity unit that accurately reflects the main activities of AA9 LPMOs and can be used to compare AA9 LPMOs of different origins.

## Methods

### Strains, plasmids, and cultivation conditions

*Escherichia coli* DH5α’ was used for plasmid construction and propagation and was cultivated in LB medium (1% tryptone, 0.5% yeast extract, 1% NaCl, supplemented with 100 μg/mL ampicillin if necessary). The *Trichoderma reesei* QM9414 strain (ATCC 26921) was used as a host for the heterologous expression of the recombinant proteins. The cultivation conditions of *T. reesei* were as previously described [33]. *Thielavia terrestris* (ATCC 38088) was cultivated on PDA agar (20% potato infusion, 2% dextrose, 2% agar) at 45 °C. Plasmid pUC19 was used for the construction of vectors or expression cassettes. Plasmid pAN7-1 containing the hygromycin B resistant cassette was used as an assisting plasmid for *T. reesei* transformation [40].

### Enzymes and chemicals

Phosphoric acid swollen cellulose (PASC) was prepared with Avicel PH-101 (Sigma–Aldrich) as the raw material following the method of Cannella et al. [41]. Horseradish peroxidase (HRP) was purchased from Macklin (Shanghai), with one unit of enzyme activity defined as the amount of enzyme necessary to form 1.0 mg purpurogallin from pyrogallol in 20 s at a pH of 6.0 and 20 °C. Ascorbate oxidase was purchased from Solarbio (Beijing), with one unit of enzyme activity defined as the amount of enzyme necessary to oxidize 1.0 μmol of L-ascorbate to dehydroascorbate per min at 25 °C. 4-Amino-antipyrine (4-AAP) was purchased from Macklin (Shanghai). 3,5-Dichloro-2-hydroxybenzenesulfonic acid (DCHBS) was purchased from Solarbio (Beijing). Other chemicals were of analytical grade and purchased from BBI Life Sciences Corporation (Shanghai).

### Construction of the expression vector and protoplast transformation

The genomic DNA of *T. reesei* and *T. terrestris* was extracted using a fungal genomic DNA extraction kit (Sangon Biotech, Shanghai). The promoter and terminator sequences of the *pdc* gene were PCR amplified from the genomic DNA of *T. reesei* QM9414 as a template. The coding sequence of GOOX from *Sarocladium strictum* was codon-optimized for expression in *T. reesei* (Additional file 1: Table S2) and was commercially synthesized by IGEbio (Guangzhou, China), with the addition of the signal peptide sequence of *T. reesei cbh1* at the N-terminus and a 6 × histidine tag at the C-terminus. The TtAA9F and TtAA9G genes (GenBank sequence numbers: THITE-2142696 for TtAA9F and THITE-170174 for TtAA9G), including their native signal peptide sequences, were PCR amplified from the genomic DNA of *T. terrestris*, and a 6 × histidine tag was fused with the C-terminus of each gene during PCR amplification. The expression cassettes were constructed by the sequential ligation of the *pdc* promoter, the target genes, and the *pdc* terminator into pUC19 with a ClonExpress™ II One Step Cloning Kit (Vazyme Biotech, Nanjing, China). For the protoplast transformation of *T. reesei*, the linearized constructs and pAN7-1 were cotransformed using the polyethylene glycol method described in Punt et al. [40].

### Recombinant protein expression and purification

The recombinant protein production in *T. reesei* was performed as described in Li et al. [33]. The total protein concentration was determined with a standard Bradford reagent kit (Sangon Biotech, Shanghai, China). Sodium dodecyl sulfate–polyacrylamide gel electrophoresis (SDS–PAGE) was performed using 10% polyacrylamide gel slabs. The protein band was visualized by staining with Coomassie Brilliant Blue G250, and the molecular weight of the observed proteins was estimated according to a prestained broad-range protein standard marker (Thermo Scientific PageRuler Prestained Protein Ladder). The expression of the target proteins was verified by Western blotting with anti-6 × His tag mouse monoclonal antibody and HRP-conjugated rabbit anti-mouse IgG (BBI Life Sciences Corporation, Shanghai). The recombinant protein productivity was analysed through densitometric quantitation of protein bands of the SDS**–**PAGE using Image J software (National Institutes of Health, USA), and was normalized to BSA loading controls. The recombinant proteins were purified by an ÄKTA Purifier UPC100 FPLC-System (GE Healthcare) as follows. The supernatant was filtered with a 0.45 μm pore polyvinylidene fluoride (PVDF) membrane to remove residual insoluble substances. The filtrate was placed in a dialysis bag with a 7000 Da molecular weight cutoff, covered with PEG 20,000 powder, and incubated at 4 °C for 12 h for concentration. The concentrated sample was dialyzed against affinity chromatography binding buffer (20 mM sodium phosphate, pH 8.0, 500 mM NaCl) for 12 h, during which the buffer was changed once. After dialysis, the sample was filtered with a 0.22 μm pore PVDF membrane, and the recombinant proteins were purified using Ni Sepharose 6 Fast Flow on the FPLC system. The flow rates of eluent A (20 mM sodium phosphate, pH 8.0, 500 mM NaCl) and eluent B (eluent A + 500 mM imidazole) were adjusted to fulfil the gradient elution condition, i.e., in the loading stage, the imidazole concentration was maintained at 25 mM; in the washing stage, the imidazole concentration was adjusted in a stepwise gradient (50**–**75 mM) to remove any nonspecifically bound proteins; and then, the imidazole concentration in the elution buffer was maintained at 250 mM imidazole to elute the target proteins. The fractions containing the purified protein were concentrated by Amicon Ultra Centrifugal Filters (Millipore, Germany) and were further purified by gel filtration chromatography using a Superdex 200 Increase 10/300 GL column (GE Healthcare) and eluted 20 mM Tris (pH 7.5) and 150 mM NaCl at a flow rate of 0.5 ml /min. To recover the possible loss of Cu^2+^ during purification, the purified LPMO solutions were mixed with an equal volume of copper sulfate solution at a concentration of 3 times the LPMOs and incubated for 1 h at 4 °C. The excessive copper was removed by desalting the protein using Sephadex G-25 Medium (GE Healthcare).

### Setting up the SsGOOX-based HRP colorimetric method for the cellobiose concentration assay

The amounts of 4-AAP and DCHBS used for the assay were first optimized. For the optimization of the 4-AAP dosage, 20 μl 4-AAP solutions with different concentrations were mixed with an equal volume of 1 mM cellobiose and loaded on a 96-well microtiter plate. Then, the chromogenic reaction was started by the addition of 20 μl of 100 mM DCHBS (final concentration 10 mM), 20 μl of 1 μM GOOX (final concentration 100 nM), 20 µl of 500 U/ml HRP (final concentration 50 U/ml) and 100 μl of sodium phosphate buffer (100 mM, pH 7.0). A_515_ was detected after incubation at room temperature for 30 min using a Synergy MX microplate reader (BioTek, Vermont, USA). For the optimization of the DCHBS dosage, a similar process was carried out, except that the roles of 4-AAP and DCHBS were exchanged, and the concentration of 4-AAP in the reagent mixture was set at 0.1 mM.

The standard curve for the cellobiose analysis was determined using a series of cellobiose solutions with concentrations ranging from 0.01 to 0.35 mM as the substrate. Twenty microlitre cellobiose solutions were loaded on a 96-well microtiter plate, and 180 μL of reagent mixture consisting of 20 μl of 1 mM 4-AAP (final concentration 0.1 mM), 20 μl of 20 mM DCHBS (final concentration 2 mM), 20 μl of 1 μM GOOX (final concentration 100 nM), 20 µl of 500 U/ml HRP (final concentration 50 U/ml) and 100 μl of sodium phosphate buffer (100 mM, pH 7.0) was added to each well to start the chromogenic reaction. A_515_ was then continuously detected at room temperature by a Synergy MX microplate reader. An assay with 20 μl pure water substituted for the cellobiose solution was used as a blank. The relation between A_515_ and the cellobiose concentration was analysed, and the standard curve was plotted in the proper cellobiose concentration range.

### Characterization of the enzymatic properties of recombinant SsGOOX

SsGOOX was characterized by measuring its catalytic reaction velocity under different conditions. The HRP colorimetric assay on the 96-well microtiter plates was used to measure hydrogen peroxide production and the reaction velocity [21]. The HRP reaction mixture contained 0.1 mM 4-AAP, 2 mM DCHBS, 50 U/ml HRP, and 50 mM phosphate buffer (pH 7.0). To determine the optimal reaction temperature, 100 nM recombinant SsGOOX was used to oxidize 1 mM cellobiose in 40 mM Britton**–**Robinson buffer (pH 7.0) for 2 min at different temperatures [42]. Then, 20 μl of the reaction mixture was added to 180 μl of the HRP colorimetric assay mixture, and A_515_ was measured when the colour reaction proceeded to 3 min at room temperature. The optimal reaction pH was determined essentially following the above approach, except that the reaction pH was set at different values in the range of 2 to 12 using 40 mM Britton**–**Robinson buffer, with the reaction temperature set at 50 °C. The thermal stability of the enzyme was determined in triplicate by incubating 1 μM GOOX in 100 mM phosphate buffer (pH 7.0) for 0, 2, 4, 6, 8, 10, 15, 20, and 60 min at 50 °C, 55 °C, and 60 °C, respectively, and the residual enzyme activity was measured at pH 7.0 and 50 °C.

For the enzyme kinetic study, 10 nM recombinant SsGOOX and a series of cellobiose concentrations ranging from 0.02 to 5 mM were used. The initial reaction velocities were obtained by measuring the reaction product when the reaction proceeded to 30 s at room temperature and a pH of 7.0, and then data were fit using the modified Hill’s equation to determine the kinetic parameters using nonlinear least squares regression in the curve fitting tool in MATLAB©.

### MALDI–TOF–MS analysis

MALDI**–**TOF**–**MS analysis of the oligosaccharide products released by the LPMO reaction was carried out on a 5800 MALDI**–**TOF**–**MS (AB SCIEX) using 5-chloro-2-mercapto-benzothiazole (CMBT) and 2,5-dihydroxybenzoic acid (DHB) as the matrix as previously described [43]. The MS data acquisition mass range was from *m/z* 500 to 2500.

### HPAEC–PAD analysis

The supernatants of the LPMO reactions were diluted 1000 times, and 25 μL of the diluted samples were injected for HPAEC analysis on a Dionex ICS5000 + system equipped with a pulsed amperometric detector (PAD detector) and a CarboPac PA200 column (3 × 30 mm guard column followed by a 3 × 250 mm analytical column). The products of the LPMO reactions were separated on the PA200 column by gradient elution at a flow rate of 0.4 ml /min at 30 °C. The flow rates of eluent A (0.1 M NaOH) and eluent B (0.1 M NaOH + 1 M sodium acetate) were automatically adjusted to fulfil the gradient elution condition, i.e., the concentration of sodium acetate in the eluent increased from 0 to 140 mM (14 min), 140 to 300 mM (8 min), 300 to 400 mM (4 min), and was held constant at 500 mM (3 min) before re-equilibration in 0.1 M NaOH (4 min). Oligosaccharides with degrees of polymerization (DPs) ranging from DP2 to DP5 (Elicityl, Crolles, France) and their oxidation products by GOOX were used as standards.

### Removing residual Asc with ascorbate oxidase

Four microlitres of ascorbate oxidase solutions with different concentrations (0, 25, 50, 75, 125, 250, 500, 1000, 1500, 2000 U/ml) were mixed with 96 μl of 2 mM cellobiose solution and 100 μl of freshly prepared 2 mM Asc solution and incubated at room temperature for 1 min. Then, 20 μl of the reaction mixtures was added to 180 μl of the SsGOOX-based HRP colorimetric reagent, which contained 0.1 mM 4-AAP, 2 mM DCHBS, 50 mM phosphate buffer (pH 7.0), 100 nM GOOX, and 50 U/ml horseradish peroxidase preloaded on a 96-well microtiter plate. A_515_ was recorded by the microtiter plate reader. A control reaction without Asc but with ascorbate oxidase (20 U/ml in the Asc oxidizing reaction mixture) was used to verify that ascorbate oxidase alone does not affect the SsGOOX-based HRP colorimetric assay.

### AA9 LPMO activity assay

PASC was used as the substrate for LPMO reaction. The 196 μl LPMO reaction mixture consisted of 100 μl PASC suspension (90 g/L for C1 LPMO activity assay, 30 g/L for C4 LPMO), 10 μL LPMO at a certain concentration, 76 μl of 100 mM phosphate buffer (pH 7.5), and 10 μl of 20 mM freshly prepared Asc solution (final concentration 1 mM). The first three solutions were added to a 1.5 ml Eppendorf tube and preincubated on a thermomixer (Eppendorf® Thermomixer Comfort) with shaking at 50 °C for 3 min. The Asc solution was then added to start the reaction. The reaction was performed with shaking (1200 r/min) at 50 °C for 30 min. After the LPMO reaction, the Eppendorf tube was immediately placed on ice. After the addition of 4 μl ascorbate oxidase solution (final concentration 20 U/ml), the tube was vortexed on a thermomixer for 5 min at room temperature to remove residual Asc and terminate the LPMO reaction. The reaction mixture was then centrifuged at 12,000 r/min in a mini centrifuge at 4 °C for 5 min. Finally, 20 μl supernatant was added to 180 μl SsGOOX-based HRP colorimetric reagent consisting of 20 μl of 1 mM 4-AAP, 20 μl of 20 mM DCHBS, 20 μl of 1 μM GOOX, 20 μl of 500 U/ml HRP and 100 μl of sodium phosphate buffer (100 mM sodium phosphate, pH 7.0) preloaded on the wells of a microtiter plate. A515 was continuously detected for 25 min by a microtiter plate reader. Possible futile formation of H_2_O_2_ during LPMO reaction was detected in the same way except that SsGOOX solution was replaced by heat deactivated SsGOOX solution in the SsGOOX based HRP colorimetric reagent.

## Supplementary Information

**Additional file 1:** **Figure S1.** Effects of chromogenic substrate concentrations on A_515_ values in HRP assay. **A**: Effect of 4-AAP concentrations on A_515_ OD values, in which the DCHBS concentration was set at 10.0 mM; **B**: Effect of DCHBS concentrations on A_515_ OD values, in which the 4-AAP concentration was set at 0.1 mM. In both of the experiments, 0.1 mM cellobiose was used as substrate, and 100 nM recombinant SsGOOX and 50 U/ml HRP were used as catalysts. Error bars show standard deviation (n = 3; independent experiments). **Table S1.** The correlated parameters of the modified Hill’s model. **Figure S2.** Effect of pH and temperature on recombinant SsGOOX activity. **A**: Enzyme activity at different temperatures. The activity was assayed at different temperatures in Britton-Robison buffer solution at pH 7.0. The enzyme activity at 50^o^C was defined as 100%. **B**: Enzyme activity at different pH values. The activity was assayed in Britton-Robison buffer solutions at different pH values (from pH 2.0 to pH 12.0), at 50. The enzyme activity at pH 9.0 was defined as 100%. Error bars show standard deviation (n = 3; independent experiments). **Figure S3.** Effect of temperature on SsGOOX stability. **A**: The residual activity of SsGOOX after incubation at different temperatures for 1 h. **B**: The residual activity of SsGOOX after incubation at 60. for different time intervals. Error bars show standard deviation (n = 3;independent experiments). **Table S2.** The DNA sequence of SsGOOX after codon optimization. **Table S3.** Links of TtAA9 genes in NCBI database. **Figure S4.** HPAEC-PAD analysis of the cello-oligosaccharide mixture standard before and after oxidation by SsGOOX. Lower line: analysis of the cello-oligosaccharide mixture (G1 to G5); upperline: analysis of the GOOX oxidized cello-oligosaccharide mixture (G1 to G5). In the SsGOOX oxidation reaction, the concentration of each cello-oligasaccharides was 1mg/ml (the molar concentrations are: G1 5.55 mM, G2 2.92 mM, G3 1.98 mM, G4 1.50 mM, G5 1.20 mM), the reaction time is 25 min. Upon action of SsGOOX, cello-oligosaccharides have been completely converted within 25 min. **Figure S5.** HRP colorimetric analysis of the amount of H_2_O_2_ generated by SsGOOX oxidation towards LPMO lytic products and futile formation of H_2_O_2_ by LPMO reaction. Futile formation of H_2_O_2_ was detected using SsGOOX based HRP colorimetric assay of which SsGOOX solution was replaced by heat deactivated SsGOOX solution. **A**: TtAA9F, **B**: TtAA9G. ■: the amount of H_2_O_2_ generated by SsGOOX oxidation indicated by absorbance at 515 nm, **□**: futile formation of H_2_O_2_ by LPMO reaction indicated by absorbance at 515 nm. The futile formation of H_2_O_2_ is magnified and shown in the inset. Error bars show standard deviation (n=3; independent experiments).

## Data Availability

All data generated or analysed during this study are included in this published article or its supplementary information files.

## Abbreviations

4-AAP: 4-Amino-antipyrine
AA9: Auxiliary activities family 9
Asc: Ascorbic acid
CBM1: Cellulose-binding domain 1
DCHBS: 3,5-Dichloro-2-hydroxybenzenesulfonic acid
DP: Degree of polymerization
FAD: Flavin adenine dinucleotide
GOOX: Gluco-oligosaccharide oxidase
HPAEC: High performance anion exchange chromatography
HRP: Horseradish peroxidase
LoB: Limit of blank
LoD: Limits of detection
LPMO: Lytic polysaccharide monooxygenases
MALDI**–**TOF**–**MS: Matrix-assisted laser desorption/ionization time-of-flight mass spectrometry
PASC: Phosphoric acid swollen cellulose

## References

[1] Zhang R (2020). Functional characterization of cellulose-degrading AA9 lytic polysaccharide monooxygenases and their potential exploitation. Appl Microbiol Biotechnol.

[2] Frommhagen M, Westphal AH, van Berkel WJH, Kabel MA (2018). Distinct substrate specificities and electron-donating systems of fungal lytic polysaccharide monooxygenases. Front Microbiol.

[3] Karlsson J, Saloheimo M, Siika-Aho M, Tenkanen M, Penttila M, Tjerneld F (2001). Homologous expression and characterization of Cel61A (EG IV) of *Trichoderma reesei*. Eur J Biochem.

[4] Vaaje-Kolstad G, Westereng B, Horn SJ, Liu Z, Zhai H, Sorlie M, Eijsink VG (2010). An oxidative enzyme boosting the enzymatic conversion of recalcitrant polysaccharides. Science.

[5] Levasseur A, Drula E, Lombard V, Coutinho PM, Henrissat B (2013). Expansion of the enzymatic repertoire of the CAZy database to integrate auxiliary redox enzymes. Biotechnol Biofuels.

[6] Bissaro B, Varnai A, Rohr AK, Eijsink VGH (2018). Oxidoreductases and reactive oxygen species in conversion of lignocellulosic biomass. Microbiol Mol Biol Rev.

[7] Filiatrault-Chastel C, Navarro D, Haon M, Grisel S, Herpoel-Gimbert I, Chevret D, Fanuel M, Henrissat B, Heiss-Blanquet S, Margeot A, Berrin JG (2019). AA16, a new lytic polysaccharide monooxygenase family identified in fungal secretomes. Biotechnol Biofuels.

[8] Westereng B, Arntzen MO, Agger JW, Vaaje-Kolstad G, Eijsink VGH (2017). Analyzing activities of lytic polysaccharide monooxygenases by liquid chromatography and mass spectrometry. Methods Mol Biol.

[9] Eijsink VGH, Petrovic D, Forsberg Z, Mekasha S, Rohr AK, Varnai A, Bissaro B, Vaaje-Kolstad G (2019). On the functional characterization of lytic polysaccharide monooxygenases (LPMOs). Biotechnol Biofuels.

[10] Frommhagen M, Westphal AH, Hilgers R, Koetsier MJ, Hinz SWA, Visser J, Gruppen H, van Berkel WJH, Kabel MA (2018). Quantification of the catalytic performance of C1-cellulose-specific lytic polysaccharide monooxygenases. Appl Microbiol Biotechnol.

[11] Wang D, Li J, Wong AC, Aachmann FL, Hsieh YS (2018). A colorimetric assay to rapidly determine the activities of lytic polysaccharide monooxygenases. Biotechnol Biofuels.

[12] Frommhagen M, Mutte SK, Westphal AH, Koetsier MJ, Hinz SWA, Visser J, Vincken JP, Weijers D, van Berkel WJH, Gruppen H, Kabel MA (2017). Boosting LPMO-driven lignocellulose degradation by polyphenol oxidase-activated lignin building blocks. Biotechnol Biofuels.

[13] Wang D, Li Y, Zheng Y, Hsieh YSY (2021). Recent advances in screening methods for the functional investigation of lytic polysaccharide monooxygenases. Front Chem.

[14] Frommhagen M, van Erven G, Sanders M, van Berkel WJH, Kabel MA, Gruppen H (2017). RP-UHPLC-UV-ESI-MS/MS analysis of LPMO generated C4-oxidized gluco-oligosaccharides after non-reductive labeling with 2-aminobenzamide. Carbohydr Res.

[15] Breslmayr E, Hanzek M, Hanrahan A, Leitner C, Kittl R, Santek B, Oostenbrink C, Ludwig R (2018). A fast and sensitive activity assay for lytic polysaccharide monooxygenase. Biotechnol Biofuels.

[16] Kittl R, Kracher D, Burgstaller D, Haltrich D, Ludwig R (2012). Production of four *Neurospora crassa* lytic polysaccharide monooxygenases in *Pichia pastoris* monitored by a fluorimetric assay. Biotechnol Biofuels.

[17] Breslmayr E, Daly S, Pozgajcic A, Chang H, Rezic T, Oostenbrink C, Ludwig R (2019). Improved spectrophotometric assay for lytic polysaccharide monooxygenase. Biotechnol Biofuels.

[18] Brander S, Lausten S, Ipsen JO, Falkenberg KB, Bertelsen AB, Norholm MHH, Ostergaard LH, Johansen KS (2021). Colorimetric LPMO assay with direct implication for cellulolytic activity. Biotechnol Biofuels.

[19] Lin SF, Yang TY, Inukai T, Yamasaki M, Tsai YC (1991). Purification and characterization of a novel glucooligosaccharide oxidase from *Acremonium strictum* T1. Biochim Biophys Acta.

[20] Lee MH, Lai WL, Lin SF, Hsu CS, Liaw SH, Tsai YC (2005). Structural characterization of glucooligosaccharide oxidase from *Acremonium strictum*. Appl Environ Microbiol.

[21] Vuong TV, Vesterinen AH, Foumani M, Juvonen M, Seppala J, Tenkanen M, Master ER (2013). Xylo- and cello-oligosaccharide oxidation by gluco-oligosaccharide oxidase from *Sarocladium strictum* and variants with reduced substrate inhibition. Biotechnol Biofuels.

[22] Haddad Momeni M, Fredslund F, Bissaro B, Raji O, Vuong TV, Meier S, Nielsen TS, Lombard V, Guigliarelli B, Biaso F, Haon M, Grisel S, Henrissat B, Welner DH, Master ER, Berrin JG, Abou Hachem M (2021). Discovery of fungal oligosaccharide-oxidising flavo-enzymes with previously unknown substrates, redox-activity profiles and interplay with LPMOs. Nat Commun.

[23] Obzansky DM, Rabin BR, Simons DM, Tseng SY, Severino DM, Eggelte H, Fisher M, Harbron S, Stout RW, Di Paolo MJ (1991). Sensitive, colorimetric enzyme amplification cascade for determination of alkaline phosphatase and application of the method to an immunoassay of thyrotropin. Clin Chem.

[24] LiCata VJ, Allewell NM (1997). Is substrate inhibition a consequence of allostery in aspartate transcarbamylase?. Biophys Chem.

[25] Armbruster DA, Pry T (2008). Limit of blank, limit of detection and limit of quantitation. Clin Biochem Rev.

[26] Harris PV, Welner D, McFarland KC, Re E, Navarro Poulsen JC, Brown K, Salbo R, Ding H, Vlasenko E, Merino S (2010). Stimulation of lignocellulosic biomass hydrolysis by proteins of glycoside hydrolase family 61: structure and function of a large, enigmatic family. Biochemistry.

[27] Westereng B, Loose JSM, Vaaje-Kolstad G, Aachmann FL, Sorlie M, Eijsink VGH (2018). Analytical tools for characterizing cellulose-active lytic polysaccharide monooxygenases (LPMOs). Methods Mol Biol.

[28] Martinello F, da Silva EL (2006). Mechanism of ascorbic acid interference in biochemical tests that use peroxide and peroxidase to generate chromophore. Clin Chim Acta.

[29] Chen K, Zhang X, Long L, Ding S (2021). Comparison of C4-oxidizing and C1/C4-oxidizing AA9 LPMOs in substrate adsorption, H_2_O_2_-driven activity and synergy with cellulase on celluloses of different crystallinity. Carbohydr Polym.

[30] Bissaro B, Kommedal E, Rohr AK, Eijsink VGH (2020). Controlled depolymerization of cellulose by light-driven lytic polysaccharide oxygenases. Nat Commun.

[31] Ogunyewo OA, Randhawa A, Gupta M, Kaladhar VC, Verma PK, Yazdani SS (2020). Synergistic action of a lytic polysaccharide monooxygenase and a cellobiohydrolase from *Penicillium funiculosum* in cellulose saccharification under high-level substrate loading. Appl Environ Microbiol.

[32] Ferrari AR, Gaber Y, Fraaije MW (2014). A fast, sensitive and easy colorimetric assay for chitinase and cellulase activity detection. Biotechnol Biofuels.

[33] Li J, Wang J, Wang S, Xing M, Yu S, Liu G (2012). Achieving efficient protein expression in *Trichoderma reesei* by using strong constitutive promoters. Microb Cell Fact.

[34] Zhou X, Xu Z, He J, Li Y, Pan C, Wang C, Deng MR, Zhu H (2020). A myxobacterial LPMO10 has oxidizing cellulose activity for promoting biomass enzymatic saccharification of agricultural crop straws. Bioresour Technol.

[35] Bissaro B, Rohr AK, Muller G, Chylenski P, Skaugen M, Forsberg Z, Horn SJ, Vaaje-Kolstad G, Eijsink VGH (2017). Oxidative cleavage of polysaccharides by monocopper enzymes depends on H_2_O_2_. Nat Chem Biol.

[36] Hegnar OA, Petrovic DM, Bissaro B, Alfredsen G, Varnai A, Eijsink VGH (2019). pH-dependent relationship between catalytic activity and hydrogen peroxide production shown via characterization of a lytic polysaccharide monooxygenase from *Gloeophyllum trabeum*. Appl Environ Microbiol.

[37] Danneels B, Tanghe M, Joosten HJ, Gundinger T, Spadiut O, Stals I, Desmet T (2017). A quantitative indicator diagram for lytic polysaccharide monooxygenases reveals the role of aromatic surface residues in HjLPMO9A regioselectivity. PLoS ONE.

[38] Cheng C, Haider J, Liu P, Yang J, Tan Z, Huang T, Lin J, Jiang M, Liu H, Zhu L (2020). Engineered LPMO significantly boosting cellulase-catalyzed depolymerization of cellulose. J Agric Food Chem.

[39] Correa TLR, Junior AT, Wolf LD, Buckeridge MS, Dos Santos LV, Murakami MT (2019). An actinobacteria lytic polysaccharide monooxygenase acts on both cellulose and xylan to boost biomass saccharification. Biotechnol Biofuels.

[40] Punt PJ, Oliver RP, Dingemanse MA, Pouwels PH, van den Hondel CA (1987). Transformation of *Aspergillus* based on the hygromycin B resistance marker from *Escherichia coli*. Gene.

[41] Cannella D, Mollers KB, Frigaard NU, Jensen PE, Bjerrum MJ, Johansen KS, Felby C (2016). Light-driven oxidation of polysaccharides by photosynthetic pigments and a metalloenzyme. Nat Commun.

[42] Lipskikh OI, Korotkova EI, Barek J, Vyskocil V, Saqib M, Khristunova EP (2020). Simultaneous voltammetric determination of Brilliant Blue FCF and Tartrazine for food quality control. Talanta.

[43] Chen C, Chen J, Geng Z, Wang M, Liu N, Li D (2018). Regioselectivity of oxidation by a polysaccharide monooxygenase from *Chaetomium thermophilum*. Biotechnol Biofuels.

