# Supplementary material for: A sensitive, accurate, and high-throughput gluco-oligosaccharide oxidase-based HRP colorimetric method for assaying lytic polysaccharide monooxygenase activity
Source: Biotechnol Biofuels Bioprod. 2022 Feb 10;15:15. doi: 10.1186/s13068-022-02112-2 (PMC8830019; doi:10.1186/s13068-022-02112-2)
Supplement: Supplementary file 1 — Additional file 1: Figure S1. Effects of chromogenic substrate concentrations on A515 values in HRP assay. A: Effect of 4-AAP concentrations on A515 OD values, in which the DCHBS concentration was set at 10.0 mM; B: Effect of DCHBS concentrations on A515 OD values, in which the 4-AAP concentration was set at 0.1 mM. In both of the experiments, 0.1 mM cellobiose was used as substrate, and 100 nM recombinant SsGOOX and 50 U/ml HRP were used as catalysts. Error bars show standard deviation (n = 3; independent experiments). Table S1. The correlated parameters of the modified Hill’s model. Figure S2. Effect of pH and temperature on recombinant SsGOOX activity. A: Enzyme activity at different temperatures. The activity was assayed at different temperatures in Britton-Robison buffer solution at pH 7.0. The enzyme activity at 50oC was defined as 100%. B: Enzyme activity at different pH values. The activity was assayed in Britton-Robison buffer solutions at different pH values (from pH 2.0 to pH 12.0), at 50. The enzyme activity at pH 9.0 was defined as 100%. Error bars show standard deviation (n = 3; independent experiments). Figure S3. Effect of temperature on SsGOOX stability. A: The residual activity of SsGOOX after incubation at different temperatures for 1 h. B: The residual activity of SsGOOX after incubation at 60. for different time intervals. Error bars show standard deviation (n = 3;independent experiments). Table S2. The DNA sequence of SsGOOX after codon optimization. Table S3. Links of TtAA9 genes in NCBI database. Figure S4. HPAEC-PAD analysis of the cello-oligosaccharide mixture standard before and after oxidation by SsGOOX. Lower line: analysis of the cello-oligosaccharide mixture (G1 to G5); upperline: analysis of the GOOX oxidized cello-oligosaccharide mixture (G1 to G5). In the SsGOOX oxidation reaction, the concentration of each cello-oligasaccharides was 1mg/ml (the molar concentrations are: G1 5.55 mM, G2 2.92 mM, G3 1.98 mM, G4 1.50 mM, G5 1.20 mM), t [file 13068_2022_2112_MOESM1_ESM.pdf]

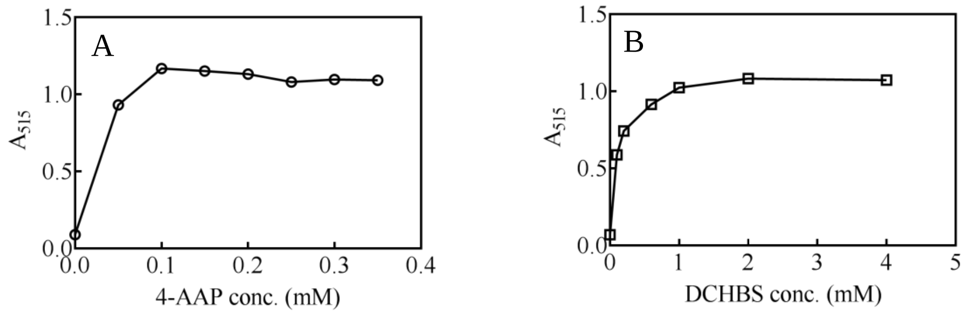

Figure S1 Effects of chromogenic substrate concentrations on A_{515} values in HRP assay. A: Effect of 4-AAP concentrations on A_{515} OD values, in which the DCHBS concentration was set at 10.0 mM; **B:** Effect of DCHBS concentrations on A_{515} OD values, in which the 4-AAP concentration was set at 0.1 mM. In both of the experiments, 0.1 mM cellobiose was used as substrate, and 100 nM recombinant SsGOOX and 50 U/ml HRP were used as catalysts. Error bars show standard deviation (n=3; independent experiments).

Table S1 The correlated parameters of the modified Hill's model.

	V_{max} (μM min^{-1})	V_i (μM min^{-1})	K_i (mM)	K_S (mM)	k_{cat}(min^{-1})	n_H
**value**	7.29	4.38	0.995	0.0177	729	0.957
**95% CI**	(6.37, 8.20)	(4.09, 4.68)	(0.543, 1.45)	(0.0119, 0.0234)	(637, 820)	(0.638, 1.28)

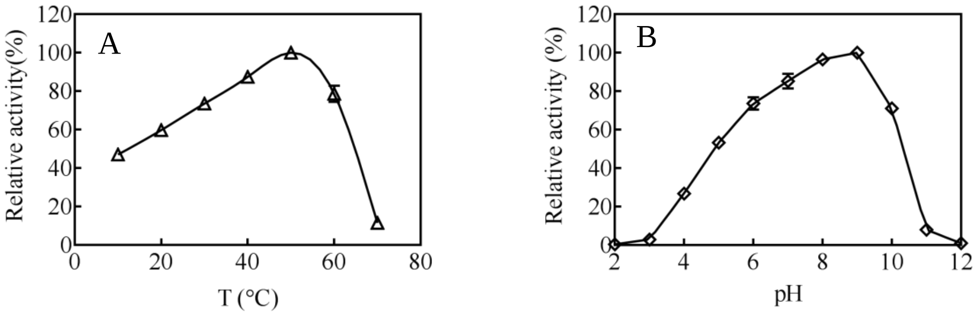

Figure S2 Effect of pH and temperature on recombinant SsGOOX activity. A: Enzyme activity at different temperatures. The activity was assayed at different temperatures in Britton-Robison buffer solution at pH 7.0. The enzyme activity at 50℃ was defined as 100%. **B:** Enzyme activity at different pH values. The activity was assayed in Britton-Robison buffer solutions at different pH values (from pH 2.0 to pH 12.0), at 50℃. The enzyme activity at pH 9.0 was defined as 100%. Error bars show standard deviation (n=3; independent experiments).

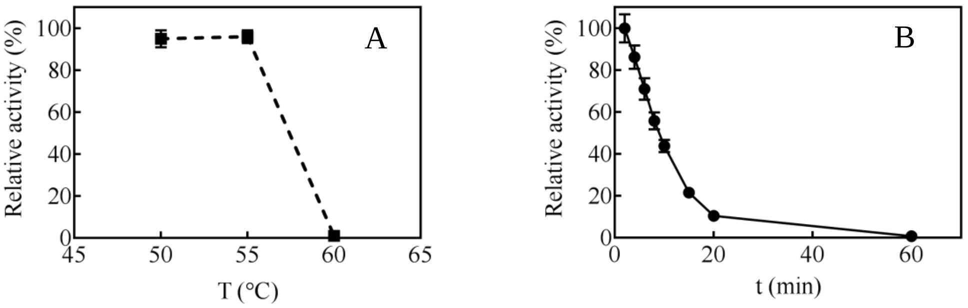

Figure S3 Effect of temperature on SsGOOX stability. A: The residual activity of SsGOOX after incubation at different temperatures for 1 h. **B:** The residual activity of SsGOOX after incubation at 60℃ for different time intervals. Error bars show standard deviation (n=3; independent experiments).

Table S2 The DNA sequence of SsGOOX after codon optimization

Enzyme	Codon optimized DNA sequence
**SsGOOX**	ATGTACCGCAAGCTGGCTGTCATTTCGGCTTTCCTCGCGACTGCCCGAGCGAACAGCATCAACGCGTGCCTCGCCGC<br>CGCTGACGTGGAGTTCCACGAGGAGGACTCGGAAGGTTGGGACATGGATGGCACTGCGTTCAACCTCCGAGTGGA<br>TTACGATCCCGCTGCGATCGCTATCCCTCGAAGCACGGAGGATATTGCCGCGGCCGTCCAGTGCGGTCTGGACGCCG<br>GTGTCCAGATCTCCGCGAAGGGAGGCGGCCACTCCTACGGCTCCTACGGCTTTGGTGGCGAAGACGGACACCTGAT<br>GCTCGAGCTGGACCGCATGTATCGCGTTTCCGTCGATGACAACAACGTCGCGACTATCCAGGGCGGAGCTCGCCTG<br>GGTTACACCGCCCTGGAACTGCTCGACCAGGGCAACCGAGCGCTGAGCCACGGTACGTGCCCTGCTGTCGGCGTC<br>GGAGGCCACGTCCTGGGAGGAGGCTACGGTTTCGCGACCCACACGCACGGCCTCACGCTGGACTGGCTGATCGGC<br>GCCACTGTGGTGCTGGCTGATGCCTCCATTGTGCATGTCTCCGAGACGGAGAACGCCGACCTCTTTTGGGCCCTCCG<br>CGGTGGAGGTGGCGGCTTCGCTATTGTCAGCGAGTTTGAGTTTAATACCTTCGAAGCTCCCGAGATCATCACCACGT<br>ACCAGGTCACCACCACCTGGAACCGCAAGCAGCACGTTGCTGGCCTGAAGGCGCTGCAGGACTGGGCCCAGAATA<br>CGATGCCTCGCGAGCTGTCGATGCGCCTCGAGATCAACGCCAACGCTCTCAATTGGGAGGGAAACTTCTTCGGCAA<br>CGCCAAGGACCTGAAGAAGATCCTCCAGCCGATCATGAAGAAGGCCGGAGGAAAGAGCACGATTTCGAAGCTCGT<br>TGAGACGGACTGGTATGGCCAAATTAACACCTACCTGTACGGCGCCGACCTGAACATTACGTACAATTATGATGTCCA<br>CGAATACTTCTACGCGAATAGCCTGACGGCTCCTCGCCTGTCCGATGAAGCGATCCAGGCTTTTGTTGACTACAAGTT<br>CGACAACTCCTCCGTCCGACCTGGCCGCGGATGGTGGATCCAGTGGGATTTCCACGGCGGCAAGAACTCCGCCCTC<br>GCTGCCGTGTCGAACGACGAGACGGCCTACGCTCACCGCGACCAGCTCTGGCTCTGGCAGTTCTATGACAGCATCTA<br>TGACTACGAGAACAACACCTCGCCGTACCCGGAGAGCGGCTTCGAGTTCATGCAAGGCTTCGTCGCCACCATTGAG<br>GATACTCTCCCTGAAGACCGCAAGGGCAAGTACTTTAACTACGCCGATACCACCCTGACCAAGGAGGAGGCCCAGA<br>AGCTGTACTGGCGCGGAAACCTCGAAAAGCTCCAGGCCATTAAGGCCAAGTACGACCCTGAAGACGTCTTTGGCAA<br>CGTGGTCAGCGTTGAGCCTATCGCCCACCATCACCACCACCAC

Table S3 Links of TtAA9 genes in NCBI database

Gene symbol	Link
**THITE_2142696**	https://www.ncbi.nlm.nih.gov/gene/11518931
**THITE_170174**	https://www.ncbi.nlm.nih.gov/gene/11517709

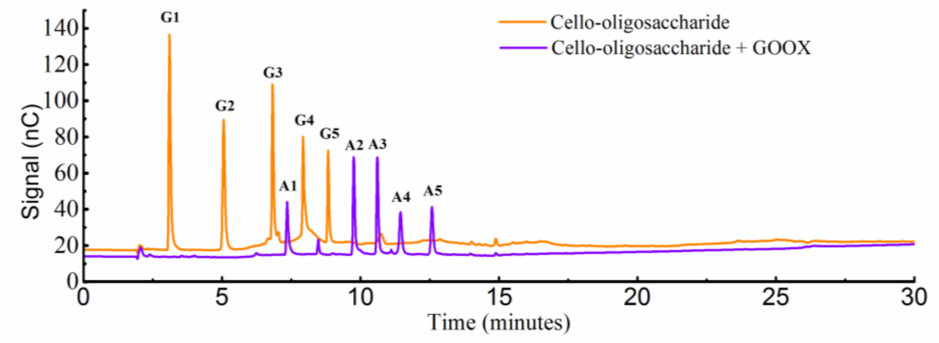

Figure S4 HPAEC-PAD analysis of the cello-oligosaccharide mixture standard before and after oxidation by SsGOOX. Lower line: analysis of the cello-oligosaccharide mixture (G1 to G5); upper line: analysis of the GOOX oxidized cello-oligosaccharide mixture (G1 to G5). In the SsGOOX oxidation reaction, the concentration of each cello-oligasaccharides was 1 mg/ml (the molar concentrations are: G1 5.55 mM, G2 2.92 mM, G3 1.98 mM, G4 1.50 mM, G5 1.20 mM), the reaction time is 25 min. Upon action of SsGOOX, cello-oligosaccharides have been completely converted within 25 min.

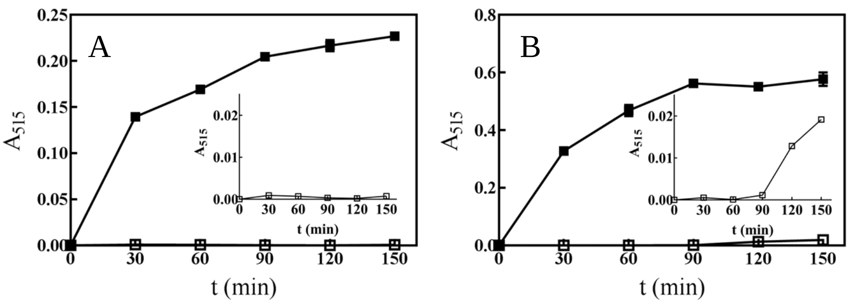

Figure S5 HRP colorimetric analysis of the amount of H_2O_2 generated by SsGOOX oxidation towards LPMO lytic products and futile formation of H_2O_2 by LPMO reaction. Futile formation of H_2O_2 was detected using SsGOOX based HRP colorimetric assay of which SsGOOX solution was replaced by heat deactivated SsGOOX solution. A: TtAA9F, B: TtAA9G. ■: the amount of H_2O_2 generated by SsGOOX oxidation indicated by absorbance at 515 nm, □: futile formation of H_2O_2 by LPMO reaction indicated by absorbance at 515 nm. The futile formation of H_2O_2 is magnified and shown in the inset. Error bars show standard deviation (n=3; independent experiments).
